# Comparative transcriptomic analysis of THP‐1‐derived macrophages infected with *Mycobacterium tuberculosis* H37Rv, H37Ra and BCG

**DOI:** 10.1111/jcmm.16980

**Published:** 2021-10-10

**Authors:** Wenyuan Pu, Chen Zhao, Junaid Wazir, Zhonglan Su, Mengyuan Niu, Shiyu Song, Lulu Wei, Li Li, Xia Zhang, Xudong Shi, Hongwei Wang

**Affiliations:** ^1^ State Key Laboratory of Analytical Chemistry for Life Science Medical School of Nanjing University Nanjing China; ^2^ Center for Translational Medicine and Jiangsu Key Laboratory of Molecular Medicine Medical School of Nanjing University Nanjing China; ^3^ Department of Dermatology the First Affiliated Hospital of Nanjing Medical University Nanjing China; ^4^ Nanjing Public Health Clinical Center the Second hospital of Nanjing Nanjing University of Chinese Medicine Nanjing China

**Keywords:** infection, macrophage, *Mycobacterium tuberculosis*, regulation, transcriptome

## Abstract

Tuberculosis (TB) remains a worldwide healthcare concern, and the exploration of the host‐pathogen interaction is essential to develop therapeutic modalities and strategies to control *Mycobacterium tuberculosis* (M.tb). In this study, RNA sequencing (transcriptome sequencing) was employed to investigate the global transcriptome changes in the macrophages during the different strains of M.tb infection. THP‐1 cells derived from macrophages were exposed to the virulent M.tb strain H37Rv (Rv) or the avirulent M.tb strain H37Ra (Ra), and the M.tb BCG vaccine strain was used as a control. The cDNA libraries were prepared from M.tb‐infected macrophages and then sequenced. To assess the transcriptional differences between the expressed genes, the bioinformatics analysis was performed using a standard pipeline of quality control, reference mapping, differential expression analysis, protein‐protein interaction (PPI) networks, gene ontology (GO) and Kyoto Encyclopedia of Genes and Genomes (KEGG) pathway enrichment analysis. Q‐PCR and Western blot assays were also performed to validate the data. Our findings indicated that, when compared to BCG or M.tb H37Ra infection, the transcriptome analysis identified 66 differentially expressed genes in the M.tb H37Rv‐infected macrophages, out of which 36 genes were up‐regulated, and 30 genes were down‐regulated. The up‐regulated genes were associated with immune response regulation, chemokine secretion, and leucocyte chemotaxis. In contrast, the down‐regulated genes were associated with amino acid biosynthetic and energy metabolism, connective tissue development and extracellular matrix organization. The Q‐PCR and Western blot assays confirmed increased expression of pro‐inflammatory factors, altered energy metabolic processes, enhanced activation of pro‐inflammatory signalling pathways and increased pyroptosis in H37Rv‐infected macrophage. Overall, our RNA sequencing‐based transcriptome study successfully identified a comprehensive, in‐depth gene expression/regulation profile in M.tb‐infected macrophages. The results demonstrated that virulent M.tb strain H37Rv infection triggers a more severe inflammatory immune response associated with increased tissue damage, which helps in understanding the host‐pathogen interaction dynamics and pathogenesis features in different strains of M.tb infection.

## 1 INTRODUCTION

Tuberculosis (TB), caused by *Mycobacterium tuberculosis* (M.tb), is one of the top killers among infectious diseases, affecting primarily the lungs and occasionally other parts of the body, resulting in extra‐pulmonary tuberculosis. In the world's population, almost one‐third is infected with M.tb and 1.2 million TB‐related deaths, and 10 million new TB cases were estimated in the WHO global TB report in 2019.^1^

The macrophages serve as the main host cell niche for the persistence and intracellular growth of M.tb. As a pathogen transmitted through the respiratory route, M.tb was first identified to undergo phagocytosis through alveolar macrophages inside the lungs. The initial infection outcome is most often a latent status in healthy individuals, in whom the host immune response is holding the bacteria in check. Active TB develops in such individuals later in life when the immune response fails. Whereas, in a minority of infected individuals, when their immune system impaired, they can develop active TB by spreading the bacilli through infectious aerosols coughed out into the surroundings and inhaled by new hosts.^2^ M.tb can survive in the hostile microenvironment of cells by escaping host immune surveillance; in this process, the molecular details of the M.tb‐macrophage interaction continue to need to be elucidated. It has been well established that various bacterial infections can significantly influence the host cells’ transcriptional profile, thus changing the disease susceptibility.^3^ Several complex processes are involved in the various stages of infection that may benefit the survival of bacteria. In particular, during these processes, various bacterial metabolites and by‐products can have some effects; these metabolites and by‐products thus serve as a crosstalk bridge between bacteria and host cells,^4^ deciding the fate of the infected cells for the clearance of the infection or substantial bacterial replication.

Apart from the influence of the diverse immune response, the susceptibility and the consequence of M.tb infection are also due to the bacilli strains, which varied in virulence and transmissibility. Different M.tb strains provoke diverse responses from the host and ultimate results.^5^, ^6^ For example, macrophages are inclined to apoptosis when infected with avirulent M.tb, which promotes antigen presentation and clearance of the infection. In contrast, necrosis of macrophages and pathogen spreading into the surrounding tissues occurs with the virulent M.tb infection.^7^

M.tb virulent strains H37Rv and avirulent strains H37Ra are two widely used laboratory reference strains, which were obtained by William Steenken in 1935 by performing a dissociation study after cultured clinical isolated M.tb H37 parent strain on glycerol‐egg media of different pH; these two strains of M.tb show different morphology, virulence as well as genetic variations. BCG (Bacillus Calmette‐Guérin), an attenuated strain of *Mycobacterium bovis*, has been given to more than 3 billion people since 1921 and is the only M.tb vaccine available for human use.^8^ Therefore, the immunological outcome of the interactions of different M.tb strains, such as avirulent H37Ra, virulent H37Rv, and BCG, with infected host cells needs to be further addressed.

To address whether differential host responses or variances in the responses to the different strains of M.tb lead to differential disease severity, our current study aimed to investigate the immune responses induced by different genotypes strains of M.tb in macrophages by transcriptomic analysis. This will help to understand the underlying mechanisms of the virulence of this economically important pathogen.

Our findings indicated that the gene expression profiles of H37RV, H37Ra and BCG infections were significantly different. In comparison with H37Ra and BCG infections, 66 unique genes were expressed in response to H37RV M.tb infection, with 36 genes up‐regulated and 30 genes down‐regulated. Increased expression of pro‐inflammatory cytokines and chemokines was observed in H37RV‐infected macrophages; these diverse gene expression profiles were associated with altered activation of inflammation‐related signalling pathways, including STAT1, NF‐kB and MAPK; additionally, we observed that, rather than inducing cell apoptosis, H37RV infection in macrophages induces a significantly higher level of pyroptosis.

## 2 MATERIALS AND METHODS

### 2.1 Bacterial culture condition

The axenic cultures of *Mycobacterium tuberculosis* H37Rv, H37Ra and BCG strains were employed in all experiments. M.tb strains were grown routinely at 37ºC in 10 ml of 7H9 (BD Middlebrook, Difco, Sparks), supplemented with 10% of oleic acid albumin dextrose catalase (OADC) (Becton Dickinson Microbiology Systems), 0.5% Glycerol (Sigma) and 0.05% of Tyloxapol (Sigma). Growth was monitored daily by OD absorbance at 600 nm for 20 days.

### 2.2 Infection of the THP‐1 macrophage cell line

The THP‐1 cell line was maintained in medium 1640 supplemented with 10% (v/v) foetal calf serum, 2 mM glutamine, 1 mM sodium pyruvate and 0.05 mM 2‐mercaptoethanol in a humidified 5% carbon dioxide atmosphere at 37°C. The THP‐1 was differentiated for 24 h using a culture medium containing 40 ng/ml phorbol 12‐myristate 13‐acetate. The cells were then washed with fresh culture medium and incubated for 48 h. Rapidly grown *M*. *tuberculosis* H37Rv, H37Ra and BCG were pelleted (3,200 rpm, 10 min), washed twice with PBS (phosphate‐buffered saline) and re‐suspended in a THP‐1 culture medium. Differentiated THP‐1 cells were optimal conditions infected with *Mycobacterium tuberculosis* at a multiplicity of infection of 5 (MOI) for 24h.^9^ The experiments were divided into four groups, including: control, H37Rv, H37Ra, and BCG‐infected groups, respectively.

### 2.3 Macrophages RNA isolation and sequencing

The total RNA extraction was prepared from M.tb‐infected THP‐1 cells with RNA Miniprep kit reagent as described by the manufacturer Vazyme biotech. NGS analysis was performed on the BGISEQ‐500 platform by BGI Genomic Services, generating per sample on average about 6.86G reads. After filtering the reads, using HISAT clean reads were mapped to the reference genome. Averagely, 90.73% reads were mapped, and by using Bowtie2 (v2.2.5), clean reads were mapped to reference transcripts; then with RSEM (v1.2.12), the level of gene expression for each sample was measured.^10^ We used Interferome 2.0 and EnrichR for annotation of transcripts; by CIMminer, clustered image maps (CIMs) were rendered. In the NCBI’s Gene Expression Omnibus database, NGS data were deposited. The sequence outcomes for each transcript were obtained as the FPKM (fragment per kilobase of exons per million reads).

### 2.4 RNA‐seq data analysis

The raw data were defined as reads containing the low‐quality reads, high content of unknown bases and the sequence of the adaptor. To decrease data noise, these reads were removed before downstream analysis. The steps of filtering are as follow: The RNA‐seq libraries’ quality was first assessed using the Fast QC v0.11.5 software. Then, according to the following parameters, the reads were subjected to standard criteria of quality control (QC): (1) trimming and cleaning reads that aligned to primers and/or adaptors, (2) reads with over 50% of low‐quality bases (quality value ≤5) in one read, and (3) reads with over 10% unknown bases (N bases). After filtering, the remaining reads are called ‘clean reads’ and stored as FASTQ format. The present study used bioinformatics methods to identify overlapping differentially expressed genes (DEGs) in THP‐1‐derived macrophages infected by M. tb. The criteria for defining genes as DEGs were |log2 fold change| >1.0 and *p* < 0.05. Standard bioinformatics analysis including PCA, gene expression, heat map, KEGG pathway, GO component, volcano plot and Venn diagram was performed by BGI.

### 2.5 Real‐Time PCR and Western blot

Total RNA extraction was prepared from M.tb‐infected THP‐1 cells. The housekeeping gene GAPDH was used as an internal control. THP‐1 mRNA primer sequences for the target genes are listed in supplementary Table 6. Real‐time PCR for gene expression was performed from cDNA using the Power SYBR Green PCR Master Mix with ABI Viia 7 detector systems under the standard protocol in a volume of 10 μl. The relative mRNA expression of each studied gene was calculated with the comparative ΔCt method using the formula 2^−ΔΔCt^. In Western blot experiment, THP‐1 cell samples were homogenized in RIPA lysis buffer with 1% phosphatase and protease inhibitor cocktail (Sigma‐Aldrich). Furthermore, the supernatants were mixed with a 5× SDS/PAGE sample buffer. Equal amounts of proteins (20 μg) were separated by a 10% SDS/PAGE gel and then transferred to PVDF membranes (Merck Millipore). Then membranes were incubated at 4℃ overnight with specific primary antibodies p‐P65, p‐STAT1, p‐ERK, p‐JNK, Caspase‐1, Caspase‐1‐cleaved, Caspase‐3, Caspase‐3‐cleaved and GAPDH at a dilution of 1: 1000 (Cell Signaling Technology). After washing with TBST three times, membranes were incubated with a secondary antibody (Cell Signaling Technology). All membranes were incubated with the HRP substrate (Millipore) for the desired durations in order to visualize the bands. In each group, the relative density of the signalling band for the protein was compared with the band for the housekeeping gene GAPDH.

### 2.6 Statistical analysis

All data were expressed as the mean ±SD from at least three independent experiments. When the distribution was normal and there was homogeneous variance, we used the ANOVA to compare amongst three or more groups. *P*‐values of 0.05 or less were considered statistically significant. All analyses were performed with Graph Pad Prism 7.0 software (version 6.07).

## 3 RESULTS

### 3.1 Quality control of sequencing data

**FIGURE 1:**
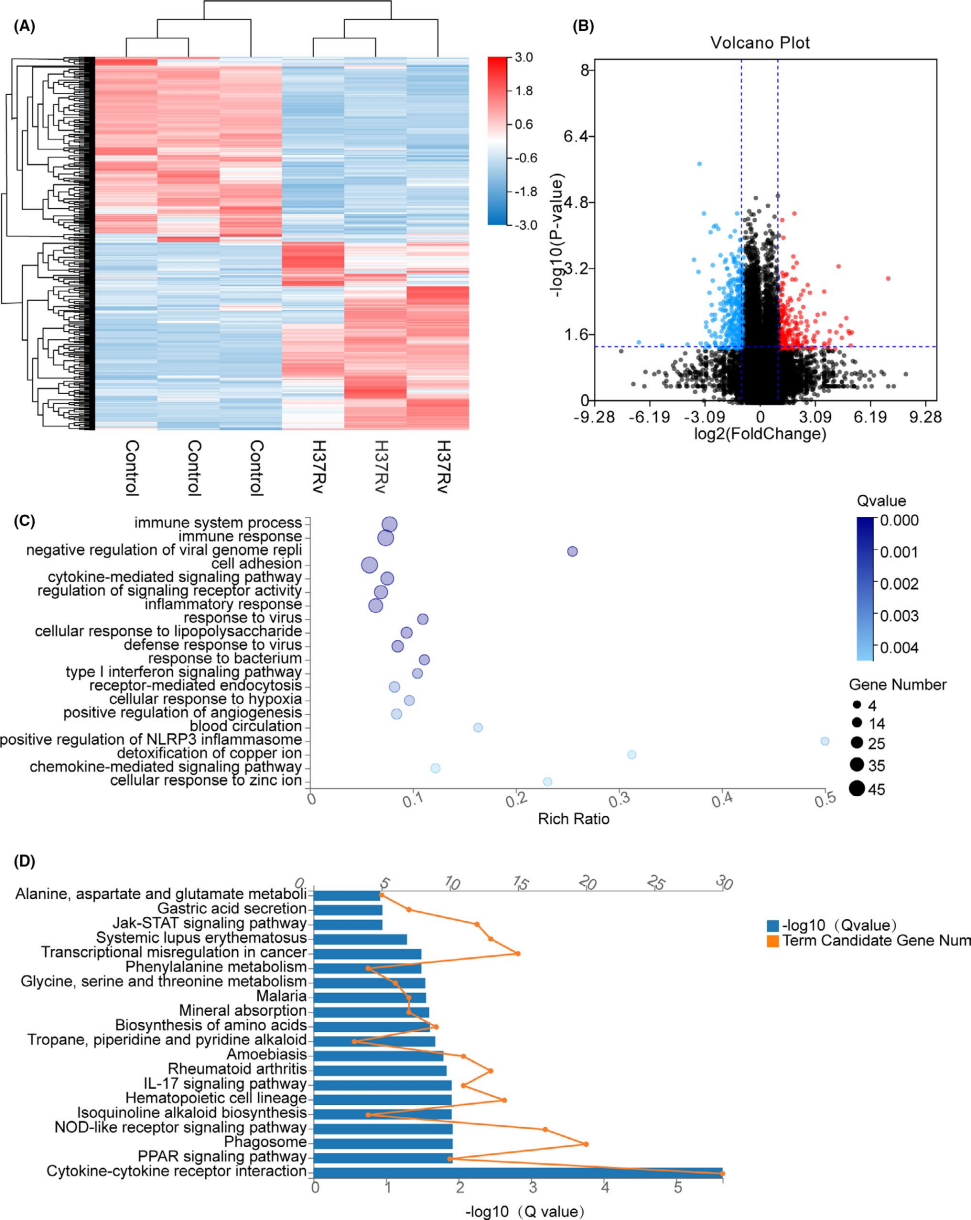
Enrichment analysis of THP‐1‐infected H37Rv strain DEGs in high‐throughput RNA sequencing. A, The heat map showing the relative transcript levels of the differential genes in THP‐1 cells uninfected and infected with H37Rv M.tb strains shows the expression pattern changes after infection. B, Volcano plot showing considerably up‐regulated (red dots) and down‐regulated genes (blue dots). C, NC vs H37Rv GO biological process enrichment analysis of significant DEGs. D, NC vs H37Rv KEGG pathway enrichment analysis of significant DEGs

The quality distribution of bases reflects the accuracy of sequencing reads. We first assessed the RNA sequencing Quality Control parameters including, sequencing yield, Q20 and Q30 (% of bases greater than Q30 is a metric for the overall quality and accuracy of the base calling of the sequencing run), Total Raw reads, Total Clean reads and Total clean bases. Overall, we can see that the ratio of bases with low quality (quality <20) is low, indicating the good quality of sequencing (Supplementary Figure 1A). The base content of each position of reads is stable, without AT or GC separation. In RNA sequencing of the Illumina platform, the 6‐bp random primers used in reverse transcription of cDNA will make the base composition of the first few positions has a preference, so the fluctuation of the first 6‐bp base proportion in the figure is a normal phenomenon. The AT and GC content of all our samples obeyed this rule (Supplementary Figure 1B). The sequencing quality of all the samples is shown in Figure 1C. We can see that the Q30 of all bases was 86.5%–87.5%, and Q20 exceeded 95%, showing that the sequencing accuracy was very high (Supplementary Figure 1C). In all the reads of our samples, the proportion of clean reads exceeded 90%, showing a high quality of sequencing (Supplementary Figure 1D).

### 3.2 General analysis of comparative transcriptome

RNA‐seq analysis of the samples from uninfected negative control and infected cells yielded an average of 45 million reads which were mapped to human genomes. Principal component analysis (PCA) revealed that the samples from uninfected macrophages clustered separately from the infected cells and the samples collected from the BCG‐infected cells as well. The samples collected from infected H37Rv and H37Ra cells were different and segregated from each other (Supplementary Figure 2A). The differential expression analysis was performed to identify these alterations. First, we compared NC vs H37Rv, NC vs BCG, NC vs H37Ra, H37Rv vs BCG, H37Rv vs H37Ra and H37Ra vs BCG six groups of gene expression profiles that included up‐ and down‐regulated number of genes (Supplementary Figure 2B). Differential expression analysis revealed that macrophage genes were significantly modulated in M.tb‐infected cells. We further performed Venn diagram analysis on 6 groups of differentially expressed genes (DEGs) (Supplementary Figure 2C). In this regard, induction of host innate immune response in macrophages can be confirmed based on the data that the numbers of DEGs were detected in each group. There were several specific or common DEGs that existed in each group, which is convenient for our next screening work.

### 3.3 Transcriptomic profile of M.tb H37Rv‐infected macrophages vs ‐uninfected Control

In total, 635 DEGs were found in the M.tb H37Rv‐infected macrophages, of which 322 genes were up‐regulated, and 313 genes were down‐regulated in the heat map (Figure 1A). Next, we performed a volcano plot analysis for the whole DEGs, which screened 103 up‐regulated genes and 169 down‐regulated genes (Figure 1B). After infection with M.tb H37Rv virulent strain, the top 10 DEGs were identified and listed in the supplementary Table 1.

To classify the functions of the DEGs, the Gene Ontology (GO) enrichment analysis was used. The DEGs were classified into three categories: biological process, cellular component and molecular function. When examining the most enriched GO terms, most of the identified DEGs were involved in the biological process category (Figure 1C). The top five terms of enrichment in the biological process category were immune system process (GO:0002376), immune responses (GO:0006955), negative regulation of viral genome replication (GO:0045071). These DEGs were also enriched in cell adhesion (GO:0007155) and the cytokine‐mediated signalling pathway (GO:0019221).

KEGG pathway enrichment analysis is useful to identify the DEGs involved in major biochemical and signal transduction pathways. Enrichment analysis of the DEGs in the M.tb H37RV‐infected versus the control groups’ pairs was performed by using the KEGG database, where the pathways that play an important role in the infection process can be further explored (Figure 1D). There were 20 pathway categories enriched in the DEGs; the top five main enrichment pathways were cytokine‐cytokine receptor interaction (KO:04060), PPAR signalling pathway (KO:03320), phagosome (KO:04145), NOD‐like receptor signalling pathway (KO:04621), and isoquinoline alkaloid biosynthesis (KO:00950).

**FIGURE 2:**
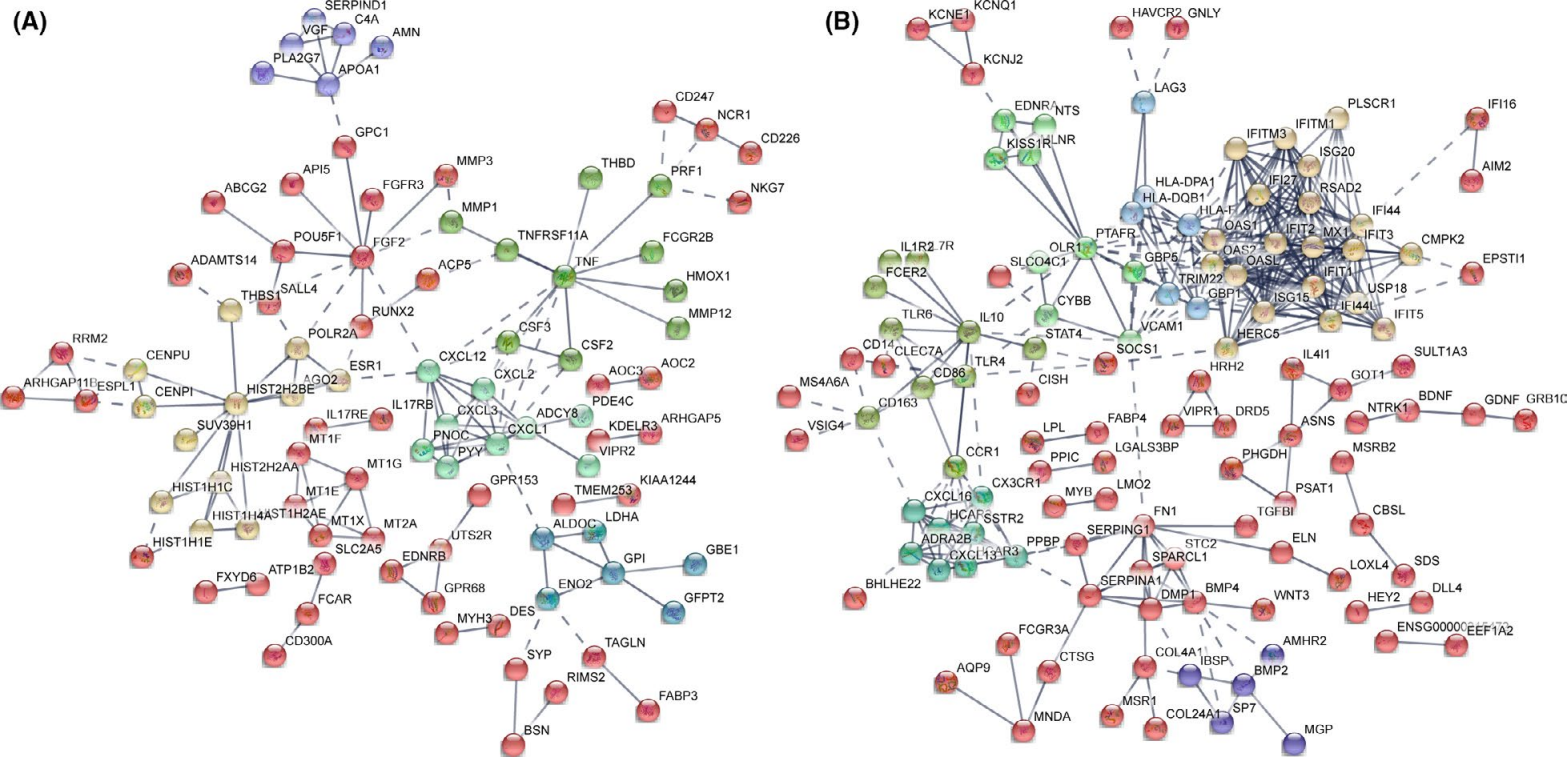
DEGs PPI networks. A, PPI network showing the up‐regulated DEGs from infected H37Rv strain group compared with the control group. B, PPI network showing the down‐regulated DEGs from infected H37Rv strains group compared with the control group. The colour representation Kmeans clustering (network is clustered into a specified number of clusters)

The protein‐protein interaction (PPI) network analysis using the Search Tool for the Retrieval of Interacting Genes (STRING) database identified an interaction network of up‐ and down‐regulated genes. In response to H37Rv infection, 322 up‐regulated genes were identified and then analysed according to node and interaction intensity. As shown in Figure 2A, TNF (degree = 11), CXCL2 (degree = 8), CSF3 (degree = 5), HIST2H2BE (degree = 11) and FGF2 (degree = 10) family proteins were activated and had a strong interaction, indicating that cells had a strong inflammatory response after infection. Likewise, we also performed a PPI analysis of 313 down‐regulated genes. As shown in Figure 2B, we found that IL‐10 (degree = 10) and other immune regulatory factors such as OAS and IFIT family proteins were the listed hub nodes within the down‐regulated DEGs, suggesting the strong infectivity and pathogenicity of H37Rv.

### 3.4 Transcriptomic profile of M.tb H37Ra‐infected macrophages vs ‐uninfected Control

**FIGURE 3:**
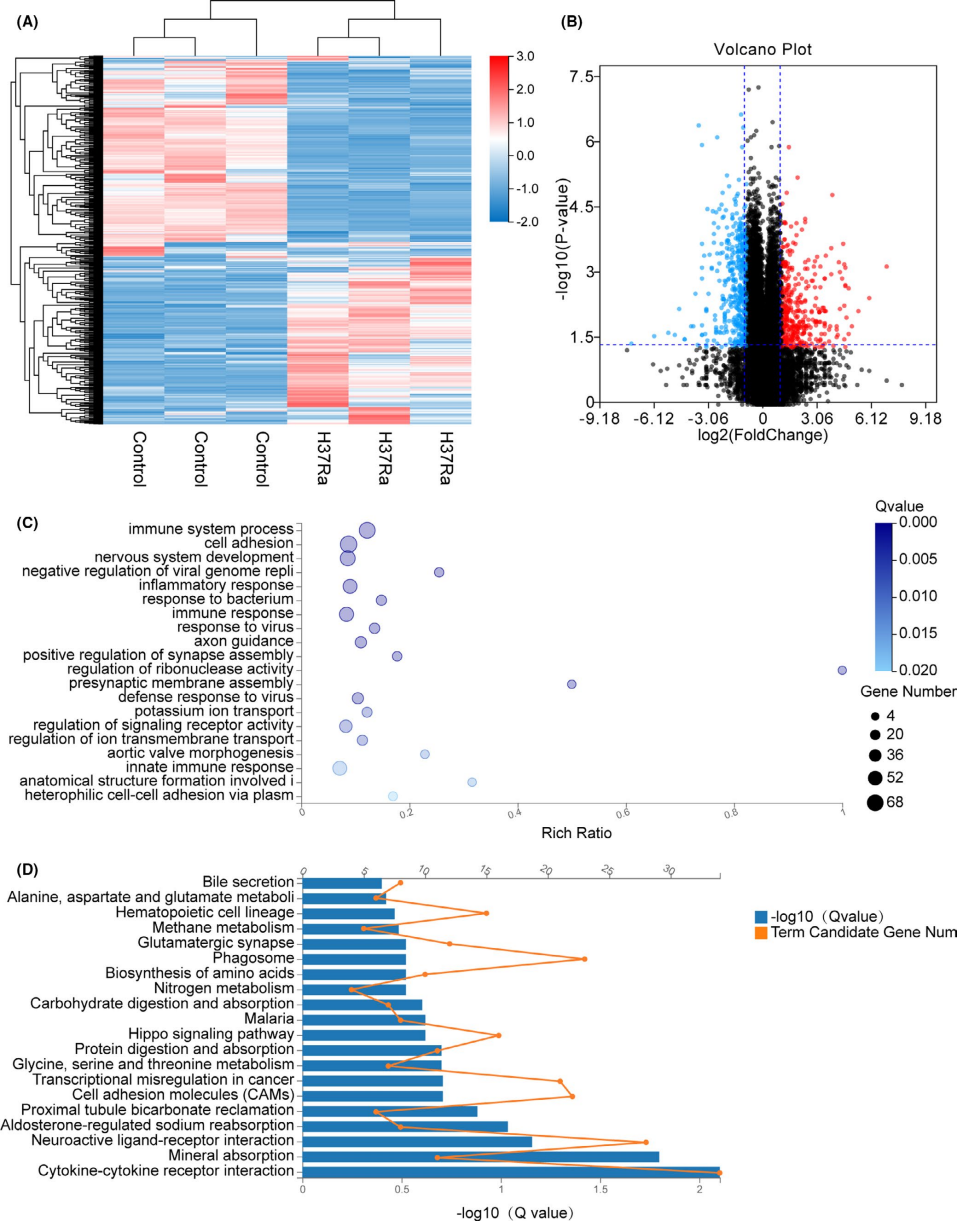
Enrichment analysis of THP‐1‐infected H37Ra strain DEGs in high‐throughput RNA sequencing. A, The heat map showing the relative transcript levels of the differential genes in THP‐1 cells uninfected and infected with H37Ra M.tb strains shows that the expression pattern changes after infection. B, Volcano plot showing considerably up‐regulated (red dots) and down‐regulated genes (blue dots). C, NC vs H37Ra GO biological process enrichment analysis of significant DEGs. D, NC vs H37Ra KEGG pathway enrichment analysis of significant DEGs

In response to M.tb H37Ra infection, we identified a total of 1041 DEGs compared with the untreated control, which included 515 up‐regulated genes and 526 down‐regulated genes in the Transcriptomic Profile analysis (Figure 3A). The volcano plot analysis for the whole DEGs screened 120 up‐regulated genes and 266 down‐regulated genes (Figure 3B); the top 10 DEGs were identified and listed in the supplementary Table 2.

Gene Ontology (GO) analysis was performed for the whole 289 DEGs; the DEGs from selected 20 pathways were examined through the GO analysis (Figure 3C). In the three main categories (biological process, molecular function and cellular component) of the GO classification, immune system process (GO:0002376), cell adhesion (GO:0007155), nervous system development (GO:0007399), negative regulation of viral genome replication (GO:0045071) and inflammatory response (GO:0006954) were dominant.

Following that, a KEGG pathway enrichment analysis was performed; 193 of the 1041 DEGs were mapped into 20 pathways (Figure 3D). Based on the KEGG function classification, these mapped DEGs were classified into a number of pathways including the following: metabolism, genetic information processing, cellular processes, organismal systems, environmental information processing and human diseases. The top five enriched altered pathways were cytokine‐cytokine receptor interaction (KO:04060), mineral absorption (KO:04978), neuroactive ligand‐receptor interaction (KO:04080), aldosterone‐regulated sodium reabsorption (KO:04960) and proximal tubule bicarbonate reclamation (KO:04964) (Figure 3D). The cytokine‐cytokine receptor interaction pathway was found to have the most genes (34 in total). Meanwhile, the KEGG pathway classification revealed that there were 107 metabolism pathways, 37 genetic information processing pathways, 323 cellular processes pathways, 215 organismal systems pathways, and 172 and 194 environmental information processing and human disease pathways, respectively.

**FIGURE 4:**
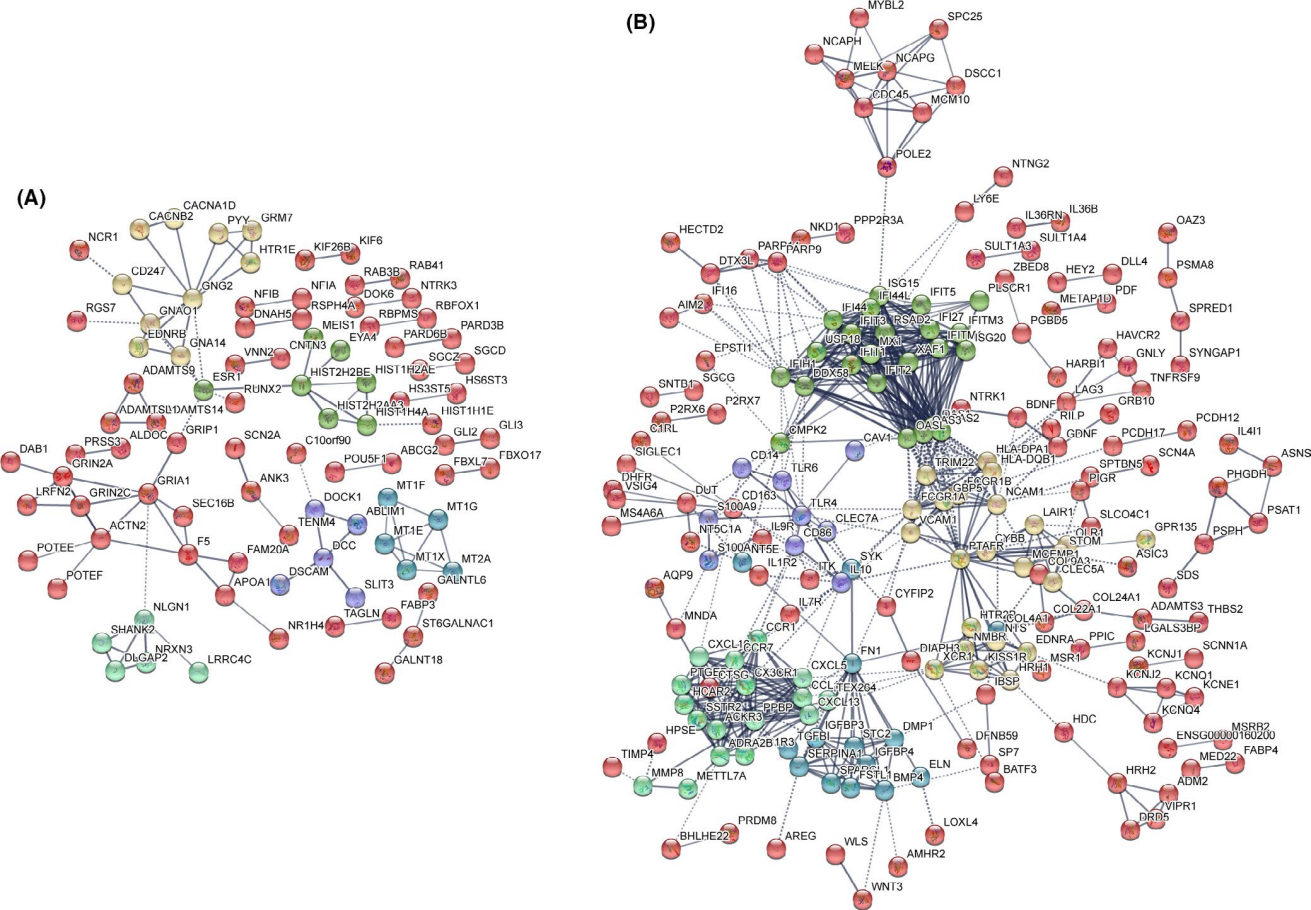
DEGs PPI networks. A, PPI network showing the up‐regulated DEGs from infected H37Ra strain group compared with the control group. B, PPI network showing the down‐regulated DEGs from the infected H37Ra strain group compared with the control group. The colour representation Kmeans clustering (network is clustered into a specified number of clusters)

Finally, we performed PPI network analysis of DEGs in the M.tb H37Ra‐infected macrophage, versus the mock infection control, to identify hub nodes. We analysed 515 up‐ and 526 down‐regulated genes by PPI and then analysed them according to node and interaction intensity. As shown in Figure 4A, in M.tb H37Ra‐infected macrophages, we identified HIST2H2BE and MT1 family proteins were up‐regulated in PPI. In addition, the PPI analysis of down‐regulated genes was also compared. According to Figure 4B, it was found that IL‐10 (degree = 10), IFIT1 (degree = 26), NCAM1 (degree = 15), CXCL5 (degree = 12), CXCL13 (degree = 8), CTSG (degree = 18) and CCR1 (degree = 12) were the listed hub nodes within the down‐regulated DEGs.

### 3.5 Transcriptomic profile of BCG‐infected macrophages vs ‐uninfected Control

**FIGURE 5:**
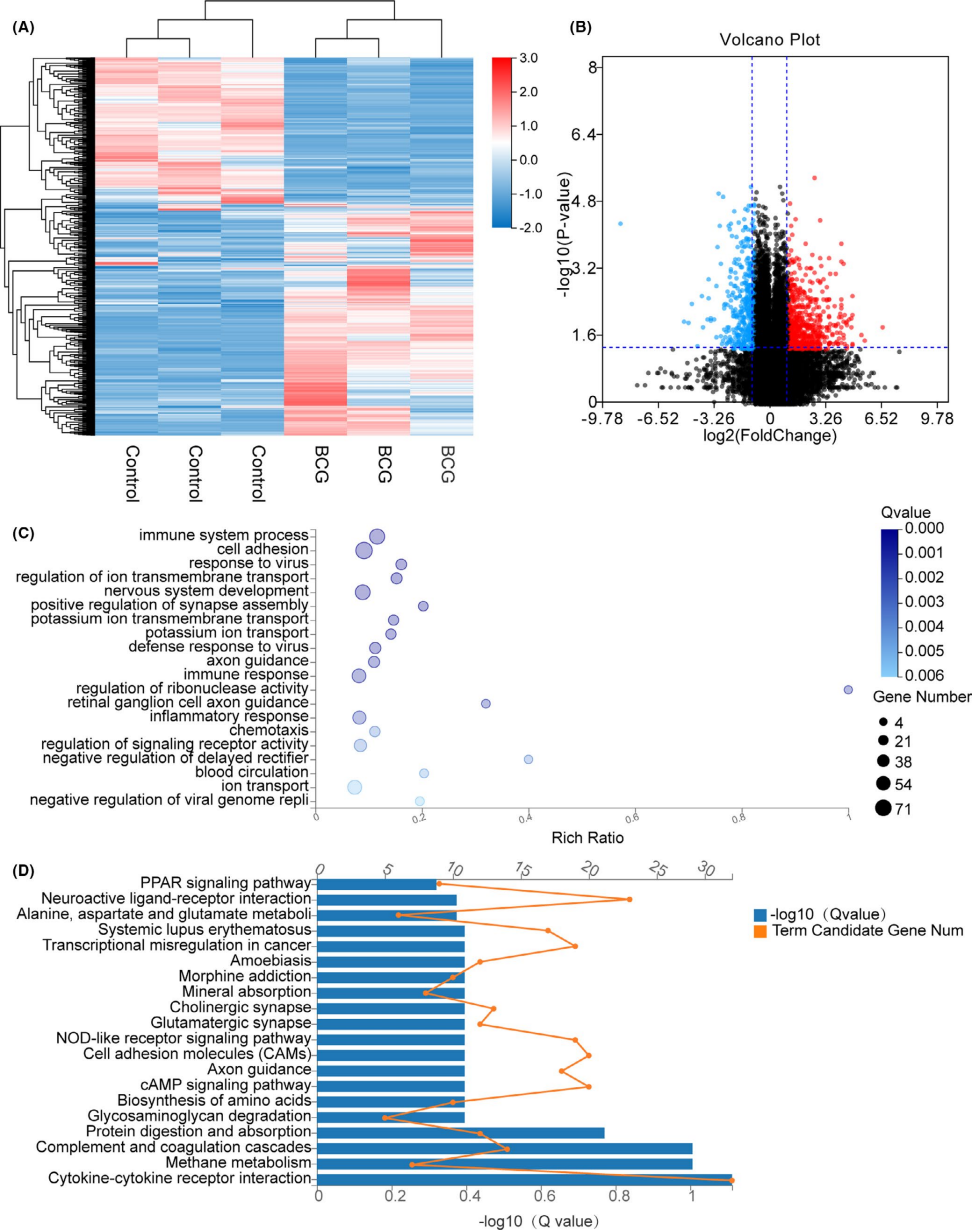
Enrichment analysis of THP‐1‐infected BCG strain DEGs in high‐throughput RNA sequencing. A, The heat map showing the relative transcript levels of the differential genes in THP‐1 cells uninfected and infected with BCG *Mycobacterium tuberculosis* strains displays the expression pattern changes after infection. B, Volcano plot showing considerably up‐regulated (red dots) and down‐regulated genes (blue dots). C, NC vs BCG GO biological process enrichment analysis of significant DEGs. D, NC vs BCG KEGG pathway enrichment analysis of significant DEGs

Our results revealed that compared with the control group, a total of 1052 DEGs were produced by the BCG‐infected group. Of them, the up‐regulated genes were 649, and the down‐regulated genes were 403 (Figure 5A). In BCG‐infected macrophages, the details of the top 10 DEGs are shown in supplementary Table 3. Volcanic maps displayed the differences in the distribution of gene expression between the BCG and the control. The genes were highlighted by the maps with an adjusted *p*‐value of <0.05 after infection and an absolute fold change of >1 (Figure 5B).

We next performed the GO Enrichment analysis; out of 321 DEGs, most of them were associated with biological processes such as the regulation of immune system processes (GO:0002376), cell adhesion (GO:0007155), response to the virus (GO:0009615), regulation of ion transmembrane transport (GO:0034765) and nervous system development (GO:0007399). In BCG‐infected groups, the 20 enriched GO terms under each category are shown in Figure 5C.

DEGs in the BCG infected macrophages were next subjected to pathway analysis. The KEGG pathway assigned 217 out of 1052 DEGs (20.62%) to 20 pathways in the KEGG database. The top enriched pathways are shown in Figure 5D. The pathways are ranked according to the enrichment score. From the KEGG pathway histogram of BCG vs Control, we can see that cytokine‐cytokine receptor interaction (KO:04060), methane metabolism (KO:00680), complement and coagulation cascades (KO:04610), protein digestion and absorption (KO:04974), and glycosaminoglycan degradation were the five most significant pathways for gene enrichment (Figure 5D).

**FIGURE 6:**
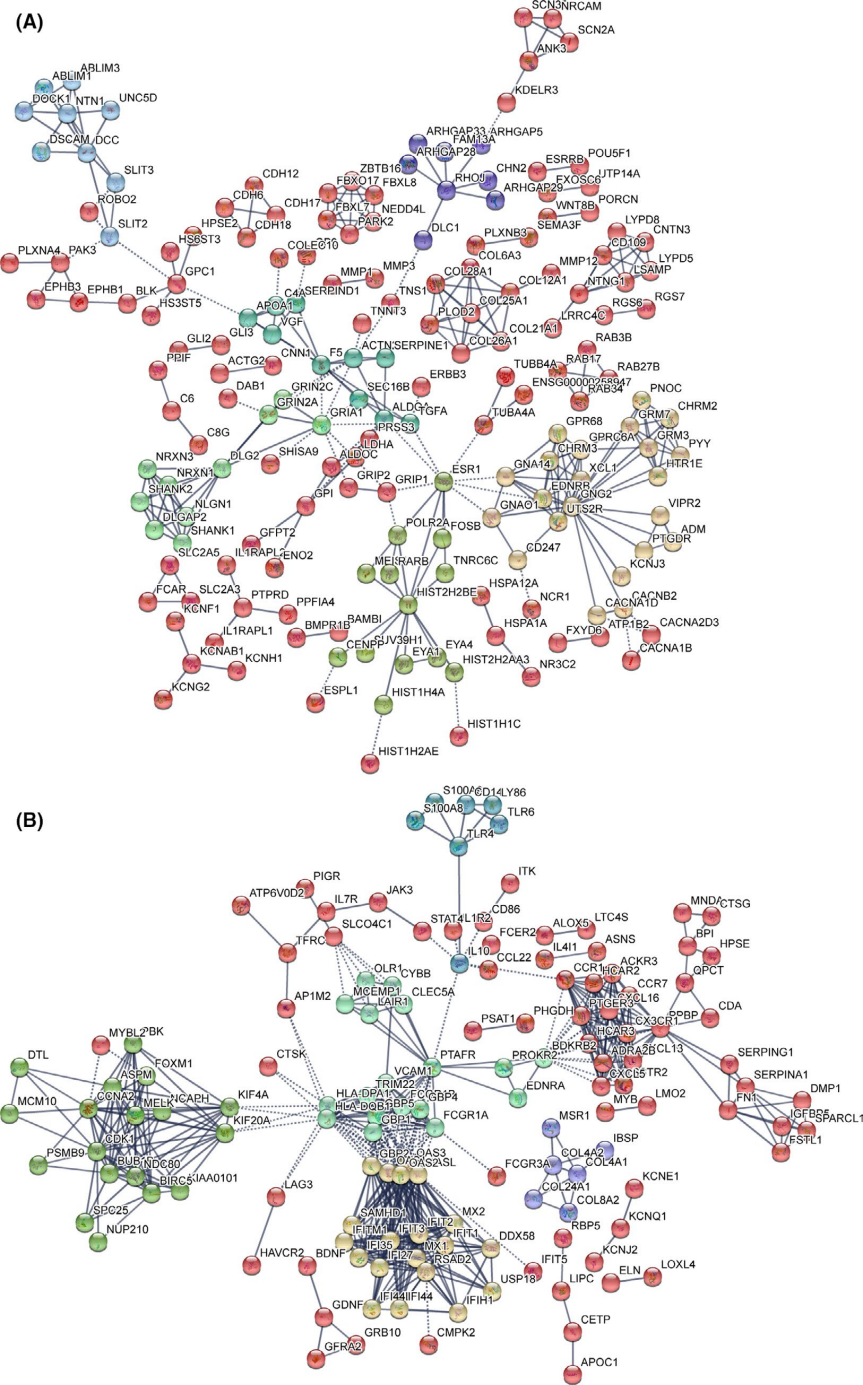
DEGs PPI networks. A, PPI network showing the up‐regulated DEGs from infected BCG strain group compared with the control group. B, PPI network showing the down‐regulated DEGs from infected BCG strain group compared with the control group. The colour representation K means clustering (network is clustered into a specified number of clusters)

To identify coherently modulated genes within the global cell signalling network, we performed a PPI network analysis of 649 up‐regulated, and 403 down‐regulated DEGs in the BCG‐infected group versus the mock control. PPI network analysis helps to reveal the flow and crosstalk between different KEGG pathways. As displaced in Figure 6, the selected network showed the signalling between cytokines and chemokines affecting T‐cell profiling and apoptosis in immune cells. According to Figure 6A, it was found that the increased expression of ESR1 (degree = 9), NTN1 (degree = 11) and TGFA (degree = 5); the decreased expression of IL17R (degree = 4), PTAFR (degree = 15), MELK (degree = 12), GBP5 NLRP3 (degree = 13), PYGER3 (degree = 19) and IFIT family proteins were the main features of the PPI network (Figure 6B).

### 3.6 Analysis of the DEGs in H37RV‐infected macrophage by comparing with H37Ra and BCG infection

**FIGURE 7:**
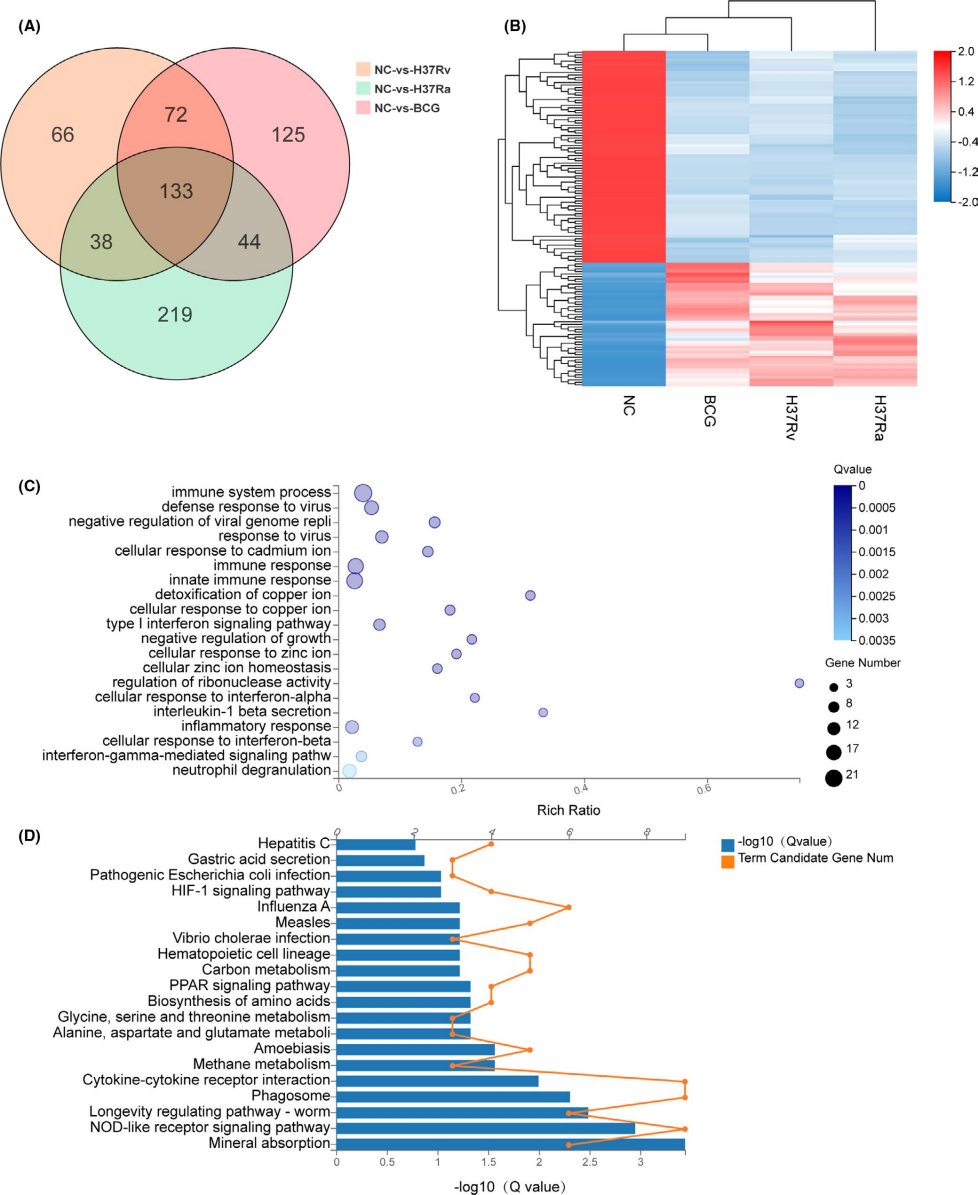
The heat map, Venn diagram, GO and KEGG classification of the DEGs screening. A, Shows the number of DEGs in three different datasets and the crossing area indicates the cross‐DEGs in different datasets. Including NC vs H37Rv (orange), NC vs H37Ra (green) and NC vs BCG (red). B, The heat map shows the relative transcript levels of the 133 differential genes in THP‐1 cells infected with *Mycobacterium tuberculosis* H37Rv, HA36Ra and BCG strains. C, Common 133 differential genes GO biological process enrichment analysis of significant DEGs. D, Common 133 differential genes KEGG pathway enrichment analysis of significant DEGs

To assess the virulent‐related factors during M.tb infection, the DEGs induced by virulent strain H37Rv infection were compared with the attenuated strain H37Ra and BCG. We performed a Venn diagram analysis of the DEGs in the various strains of M.tb‐infected groups (Figure 7A). Venn analysis showed that there were 133 common DEGs shared by all of the M.tb and BCG‐infected groups (Supplementary Table 4), and there are 72 common DEGs shared by H37Rv and BCG infected groups, 44 common DEGs shared by H37Ra and BCG‐infected groups, and 38 common DEGs shared by H37Ra and H37Rv infection groups (Figure 7A). To further characterize the change in transcription level of macrophages in various strains of M.tb infection, these 133 common DEGs were further analysed (Figure 7B). The GO and KEGG analysis showed that these DEGs were enriched in mineral absorption pathway (KO:04978), NOD‐like receptor pathway (KO:04621), phagosome pathway (KO:04145) and longevity regulating pathway (KO:04212) (Figure 7C and D).

**FIGURE 8:**
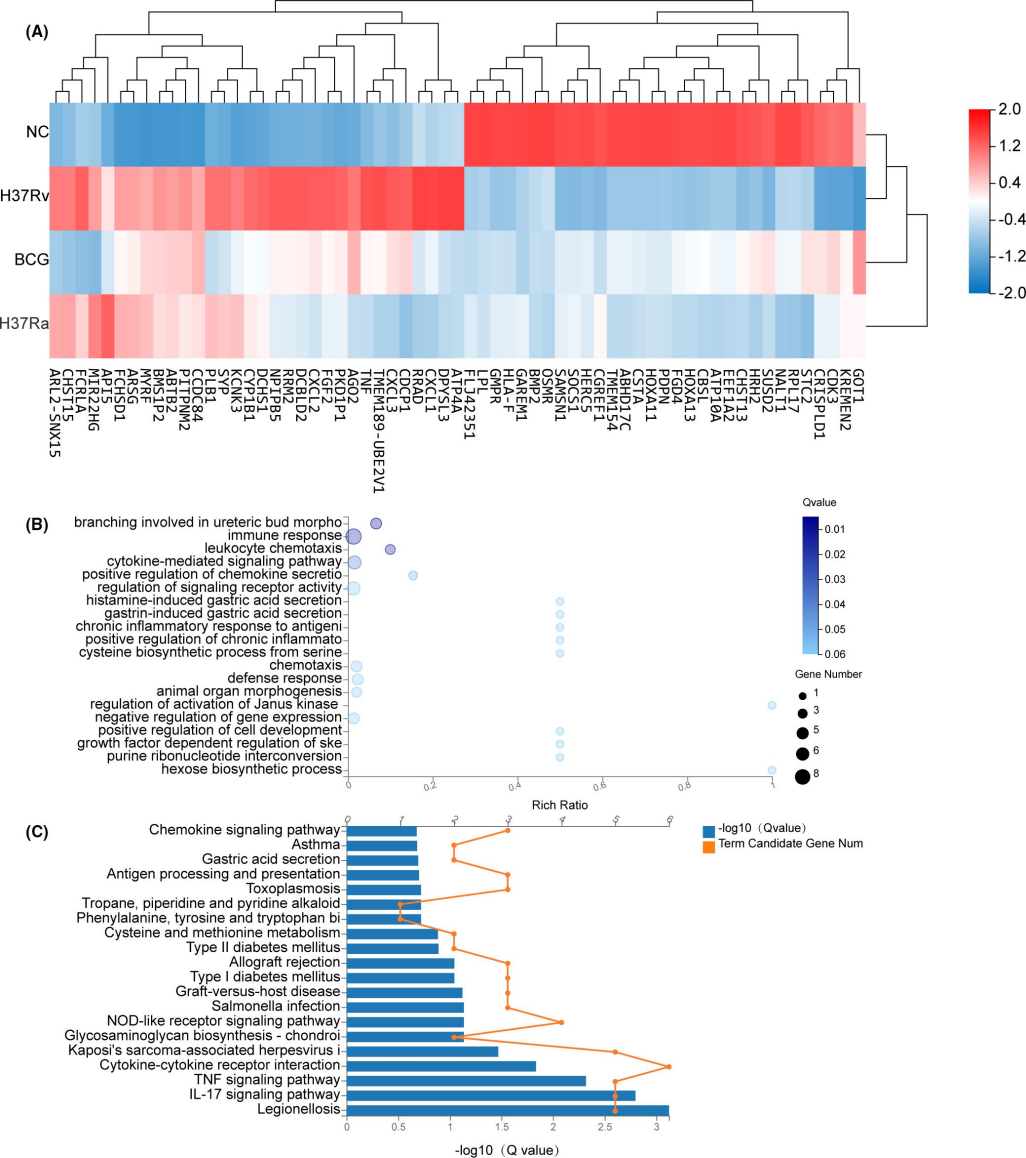
The heat map GO and KEGG of the DEGs screening. A, The heat map showing the relative transcript levels of the 66 differential genes in THP‐1 cells infected with *Mycobacterium tuberculosis* H37RV strains. B, NC vs H37Rv GO biological process enrichment analysis of 66 significant DEGs. C, NC vs H37Rv KEGG pathway enrichment analysis of 66 significant DEGs

Notably, there were 66 unique DEGs identified in the highly violent strain H37Rv‐infected group (Supplementary Table 5), (Figure 8A). The full list of significantly enriched functions from the GO Enrichment analysis is shown in Figure 8B. Among the functions significantly enriched by these DEGs were immune response, chemokine secretion and leucocyte chemotaxis, amino acid biosynthesis and energy metabolism. The identified enriched pathway included legionellosis pathway, cytokines and receptor signalling pathway, amino acid metabolism and glycosaminoglycan biosynthesis pathway, as shown in Figure 8C. The DEGs attributable to immune response, chemokine secretion and leucocyte chemotaxis process were prominent in our samples. The expression of these DEGs was mostly up‐regulated, including TNF‐a, CXCL1, CXCL2, CXCL3 and FGF2. The DEGs involved in amino acid biosynthesis and energy metabolic processes were GOT1, CBSL, SOCS1, LPL and STC2, and their expressions were largely down‐regulated in response to H37Rv infection. Additionally, we observed a significant number of DEGs involved in connective tissue development and extracellular matrix organization, including BMP2, PDPN, HOXA11 and HOXA13, whose expression was significantly down‐regulated in H37Rv‐infected macrophage.

### 3.7 Validation of DEGs and signalling pathway activation in H37RV, H37Ra and BCG‐infected macrophage

**FIGURE 9:**
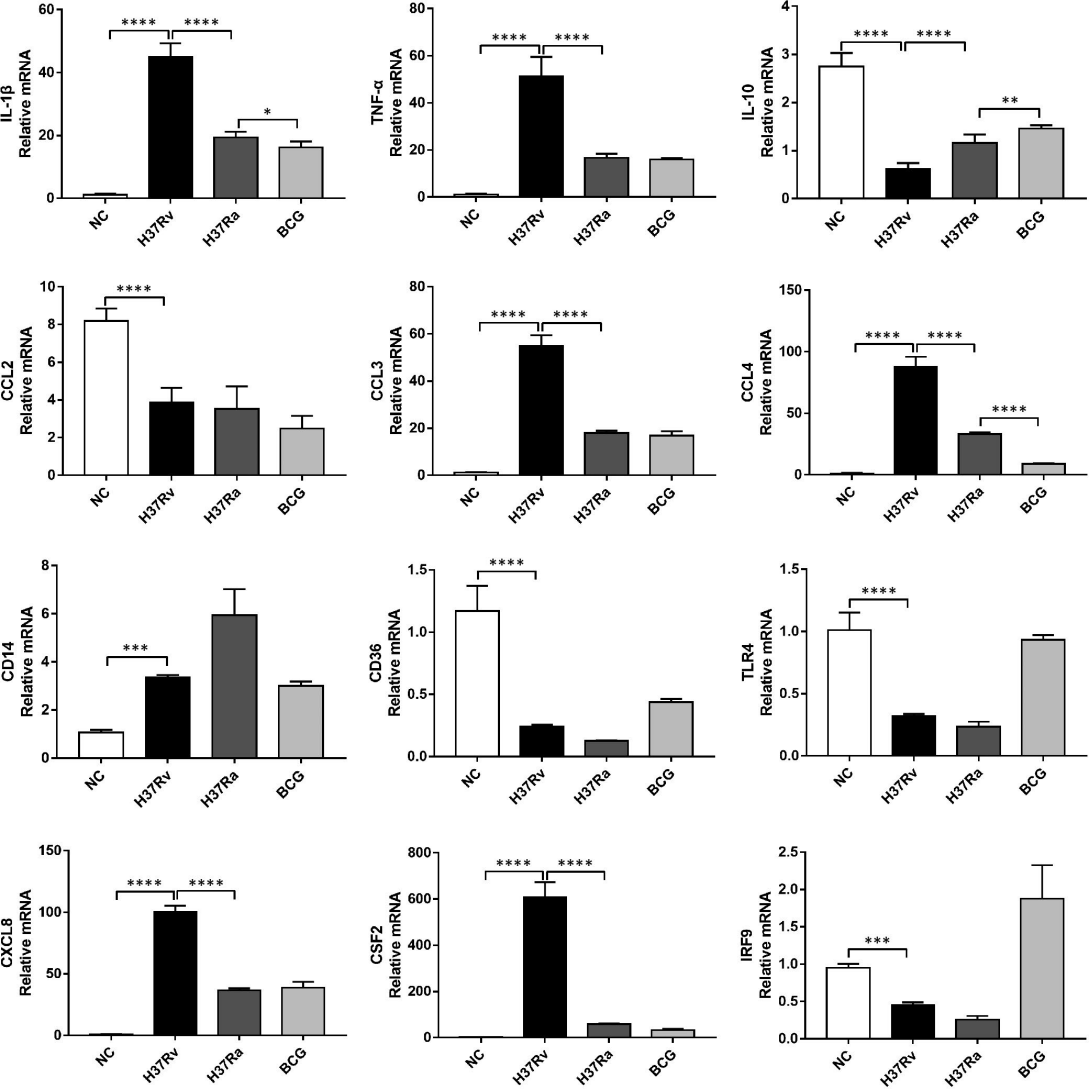
Comparative analysis of pro‐inflammatory factors and immune regulatory gene expression in different strains of MTB‐infected THP‐1 cells by Q‐PCR. H37RV, H37Ra and BCG‐infected THP‐1cell mRNA was extracted, and the expression abundance of IL‐1β, TNF‐α, IL‐10, CCL2, CCL3, CCL4, CD14, CD36, TLR4, CXCL8, CSF2 and IRF9 was determined by RT‐qPCR. The comparative analysis of transcriptional expression levels of these cytokines, chemokines and immune regulatory factors was assessed and shown in the graph. Data are expressed as the mean ± SD; *p < 0.05, **p < 0.01, ***p < 0.001, ****p < 0.0001

To verify the transcriptome analysis data, we further analysed the DEGs and signalling transduction pathway activation in H37Rv, H37Ra and BCG‐infected macrophages by Q‐PCR and Western blot. As shown in Figure 9, in Q‐PCR analysis, there was a significant increase in several pro‐inflammatory cytokines, including IL‐1β and TNF‐α in H37Rv‐infected macrophages, compared with H37Ra and BCG infection (Figure 9). Interestingly, we found that the expression of immunosuppressive factors, such as IL‐10, was significantly down‐regulated upon H37Rv infection (Figure 9). In terms of chemokine expression, CCL3, CCL4 and CXCL8 levels were significantly up‐regulated in response to H37Rv infection, whereas CCL2 levels were significantly down‐regulated (Figure 9). Other immune regulatory molecules, such as CD14 and CSF2, were significantly up‐regulated, while others, such as TLR4, IRF9 and CD36, were significantly down‐regulated after infection with H37Rv (Figure 9).

**FIGURE 10:**
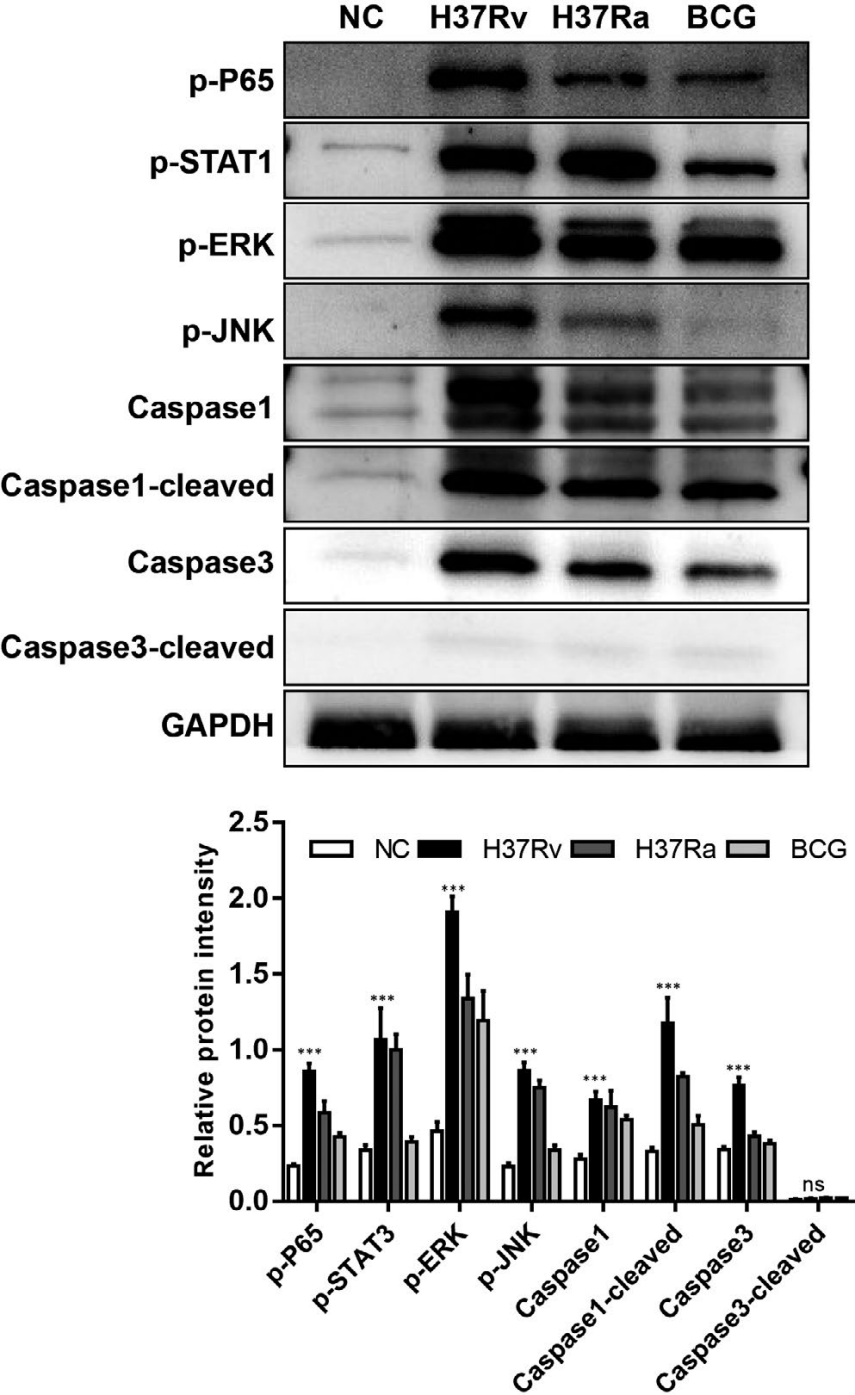
Detection of the signalling transduction pathway activation in different strains of MTB‐infected THP‐1 cells by Western blot. A, The protein expression of p‐P65, p‐STAT1, p‐ERK, p‐JNK, Caspase‐1, Cleaved‐Caspase‐1, Caspase‐3, Cleaved‐Caspase‐3 and GAPDH in different strains of MTB‐infected THP‐1 cells was assessed by Western blot. B, The graph shows the comparison of the relative intensity of the protein expression in the Western blot; the relative intensity of each band was calculated after normalization with the intensity of GAPDH in a blot, *p < 0.05, **p < 0.01, ***p < 0.001, ****p < 0.0001

In the transcriptome analysis, through GO and KEGG analysis of DEGs, we noticed that the signal pathway and cell function of cellular inflammatory response were significantly activated after infection with M.tb, especially in H37Rv. Therefore, we further validated the inflammatory‐related signalling pathway activation by measuring the pathway‐related protein expression by Western blot. As shown in Figure 10, the expression levels of p‐P65, p‐ERK and p‐JNK, which are associated with the activation of NF‐κB and MAPK related pathways, as well as the activation of STAT1, were significantly higher in H37TV infected cells compared with H37Ra or BCG infection, indicating that the virulent strain of MTB infection is likely to trigger a higher level of inflammation, which is consistent with our Q‐PCR and the transcriptome analysis data (Figure 10). Meanwhile, we investigated the mode of cell death following infection by measuring the expression of caspase‐1, cleaved‐caspase‐1, Caspase‐3 and Cleaved‐Caspase‐3. We discovered a significant increase in cleared‐caspase‐1 expression in H37RV‐infected macrophages when compared to H37Ra or BCG infection; however, the expression of Cleared‐Caspase‐3 was not detectable in all cases, indicating that pyroptosis, rather than apoptosis, occurred after virulent MTB infection (Figure 10).

## 4 DISCUSSION

The *Mycobacterium tuberculosis* complex (MTBC) consists of more than 10 different mycobacterial species that are the pathogens of tuberculosis (TB) both in humans and other species.^11^ Upon M.tb infection, the heterogenesis clinical outcome has been found due to the different virulent strains of Mtb; this heterogeneity causes greater difficulty in eradicating tuberculosis disease.^12^, ^13^, ^14^, ^15^ The understanding of host‐pathogen interaction dynamics in various virulence strains of Mtb infection will be crucial for setting up a prevention strategy for the eradication of tuberculosis disease. Therefore, in this study, we compared the transcriptional response of two well‐established M.tb laboratory strains (H37Rv and H37Ra) and a vaccine strain of M. bovis (BCG)‐infected macrophages. We identified unique transcriptional features of virulent strains of M.tb‐mediated differentially expressed genes to understand the infectious‐induced pathogenesis, which might help prevent this disease.

Macrophages play a crucial role in the immune defence against M.tb infection and are the major cell type favourably infected by M.tb. Generally, practised infection models for *in vitro* studies include the primary macrophages, mainly the mouse bone marrow‐derived macrophages (BMDMs), or the human monocyte‐derived macrophages (hMDMs) and cell lines, such as the murine J774 and RAW 264.7 or the human THP‐1 and U937. Since the primary healthy alveolar macrophages are relatively difficult to obtain due to the limited supply from the clinic, also concerning the ethical issues of human biological samples and the variation of genes that disturb the result interpretation. The use of the cell line to substitute the primary macrophages provides a promising approach for investigating the immune response and pathogenesis of TB infection *in vitro*. Murine cell lines have been found to trigger different immune responses compared with primary macrophages^16^; however, THP‐1 cells are appropriate substitutes for hMDMs for *in vitro* experiments of M.tb infection.^17^ Therefore, the THP‐1‐infected cell model was adopted in our current study.

Macrophage polarization is increasingly known as an influential pathogenesis factor in multiple types of infectious and inflammatory diseases. Classical M1 and alternative M2 polarization are the two extremes of macrophage phenotype transformation.^18^ Pro‐inflammatory M1 macrophages show anti‐infection activity by promoting T helper (Th) 1 response. M2 macrophages promote Th2 responses and contribute to tissue repair, which are supposed to be anti‐inflammatory.^19^ In accordance with previous studies,^16^ we confirmed that H37Rv infection in THP‐1‐derived macrophages induced increased TNF‐a expression and indicated the M1 polarization potential; compared with BCG or H37Ra infection, H37Rv infection selectively induced IL‐23 rather than IL‐12 in THP‐1‐derived macrophages. This result indicated that H37Rv is more likely to induce Th17‐dominated inflammatory T‐cell responses rather than Th1 responses, which play a more vital role in the elimination of intracellular pathogen infection such as M.tb. Moreover, the analysis of IL‐17 expression‐related molecules further confirms our results, since there were enhanced expressions of IL‐17RB and IL‐17RE in the H37Rv‐infected macrophages, which has not been observed in the BCG or H37Ra‐infected groups. It suggested that H37Rv infection in macrophages predominantly induces inflammatory responses. The increased expression of various cytokines and chemokines further supports the notion that inflammatory immune responses are the predominant feature of the H37Rv infection. H37Rv infection‐triggered increased inflammatory immune response is also confirmed based on the increased gene expression profiles of chemokines such as CXCL1, CXCL2, CXCL3, CXCL8, CCL3 and CXCL4 which help in the recruitment of poly‐morpho‐nuclear cells such as neutrophils during tissue inflammation. Our Q‐PCR data validate this result, and checking the inflammatory‐related signalling transduction pathway activation further confirmed this conclusion because there was enhanced activation of STAT1, NF‐kB and MAPK pathways observed in response to H37Rv infection.

Pathogen‐induced cell death is another concern associated with the pathogenesis of the disease. In macrophages, M.tb infection was the extensive activator of all currently known programme cell death (PCD) pathways, including the following: apoptosis, pyroptosis, necrosis, necroptosis, pyronecrosis, NETosis and autophagy. M.tb‐infected cells produce a potent adenylate cyclase toxin to prevent phagocytic killing, which can disrupt cell signalling in macrophages and ultimately induce apoptosis. A previous study indicated that Mtb infection could induce apoptosis via the TLR2/TLR4/MyD88/p38/ERK/PI‐3K/NF‐kB pathway. In contrast, necrosis was favoured in the absence of TLR4 signalling independently of p38, ERK1/2, PI‐3K or NF‐κB activity.^20^ Our study confirmed that there was increased activation of both extrinsic and intrinsic apoptosis pathways during M.tb infection, suggesting that M.tb infection might stimulate macrophage apoptosis. However, after measuring the expression of cleaved caspase‐3 by Western blot, we failed to detect obvious caspase‐3 activation in response to all of the MTB strain infections, suggesting that M.tb infection‐induced cell apoptosis might be time and dose dependent, but independent of the virulent nature of the M.tb strain.^21^ Pyroptosis is a programmed cell death characterized by increased activation of caspase‐1 and the generation of a vast amount of pro‐inflammatory cytokines. A previous study reported that *Mycobacterium tuberculosis* infection in macrophages could trigger plasma membrane damage which caused NLRP3 activation and pyroptosis.^22^ Our current study confirmed this finding, as there was increased expression of IL‐1β and cleaved Caspase‐1 in all M.tb strain infections, and most notably, we discovered that H37Rv infection in macrophages induced the expression of much higher levels of IL‐1β and cleaved Caspase‐1, implying that Mtb‐induced pyroptosis is associated with the virulent nature of M.tb strain, which partly explains the pathogenesis of virulent stains of Mtb infection.

Innate immune recognition, which relies on various types of pattern recognition receptors (PRRs), such as Nod‐like receptors (NLRs), Toll‐like receptors (TLRs) and C‐type lectin receptors (CLRs), plays a crucial role in recognition of M.tb. As expected, several molecules are involved in innate immune molecular recognition that has been altered or expressed in response to the various strains of M.tb infection; these include CD14, FN1 and TLR4. CD14 is an essential pattern recognition receptor protein in innate immunity, especially during M.tb infection. M.tb interacts with macrophages through cell surface receptors including CD14, and a CD14 polymorphism has been associated with M.tb infection susceptibility. FN1, which encodes fibronectin (FN), was found to be constitutively expressed in lung macrophages.^23^ M.tb infection in macrophage relies on the binding of FN with its attachment proteins, which includes the opsonization and phagocytosis process of M.tb via interactions with complement receptors and integrin receptors.^24^, ^25^ We observed increased expression of the CD14 and FN1 genes following BCG, H37Ra and H37Rv infection, confirming their critical role in M.tb infection. TLR4 is a critical component of the immune response against tuberculosis; its genetic polymorphisms have been linked to latent or active M.tb infection.^26^ In this study, we observed a significant decrease in TLR4 expression in transcriptome analysis data, which was confirmed by Q‐PCR, indicating that M.tb could down‐regulate TLR4 expression to inhibit and evade macrophage cognition.

As for the H37RV‐specific DEGs associated with pattern recognition, we detected increased expression of NOD‐like receptor in response to H37RV infection. NOD‐like receptors (NLRs), including NOD1, NOD2, NLRP3 and NLRC4, are a group of pattern recognition receptors expressed in the cell cytosol that are involved in the activation of inflammatory pathways and the recognition of microbial components that protect against invading pathogens. Its function in M.tb infection contains two aspects: the first one is its immune defence functions, as the NOD1/2 has been found to recognize and defend against M.tb infection; the second function is the activation of the inflammasome. ESAT‐6 derived from M.tb has been shown to strongly activate the NLRP3/ASC inflammasome in macrophages, correlating with airway inflammation. The increased activation of NLRs indicated that the M.tb H37RV violence strain is likely to elicit a number of severe anti‐bacterial immune responses when it infects macrophage. AGO2 is another critical gene involved in anti‐M.tb infection. Immune defence is mediated by RNA interference (RNAi); AGO2 was recently implicated in mammalian anti‐infection defence as a result of its role in miRNA biogenesis and function.^27^

In *Leishmania*‐infected macrophages to establish an anti‐inflammatory response, the expression and phosphorylated status of Ago2 protein regulated miRNA‐targeted pro‐inflammatory cytokines.^21^ As for M.tb infection, single nucleotide polymorphisms (SNPs) of AGO2 have also been found linked with tuberculosis susceptibility in the Chinese Uygur population.^28^ Here, we detected increased expression of the AGO2 gene in H37Rv‐infected macrophages, further supporting its anti‐M.tb function.

Altered expression of metabolic function genes is another feature of M.tb infection. We found several common metabolic‐associated genes with HOMX. Induction of HO1 through M.tb infection has been found to be associated with inflammation and bacterial growth. HO1 is an enzyme that regulates the expression of carbon monoxide, which was sensed by M.tb. HO1 catalyses the breakdown of haeme into carbon monoxide, biliverdin and iron. In the M.tb‐infected animal model, mice deficient in HO1 are more vulnerable to the infection of M.tb compared with wild‐type mice, which is likely due to the cytotoxic effects of free haeme accumulation.^29^ Here, we detected the enhanced expression of HO1 in response to the various strain of M.tb infection suggested that the HO1‐regulated carbon monoxide synthesis might be important for M.tb and replication in macrophage, which in agree with the report that chemical inhibition of HO1 both decreases the production of inflammatory cytokine and restricts the mycobacteria growth in infected macrophages.^30^

CD36 is a scavenger receptor expressed in multiple cell types, including macrophages. Its function involves lipid metabolism, immune responses, molecular adhesion and apoptosis. Since Mtb could utilize host‐derived lipids as a source of nutrition during infection, patients with Mtb infection develop pronounced disorders of lipid metabolism.^31^ Previous studies have demonstrated that the function of CD36 is involved in the infectivity of Mtb. CD36‐mediated lipids uptake in macrophages promotes intracellular growth of Mtb^32^ Conversely, CD36 deficiency attenuates experimental mycobacterial infection.^31^, ^33^ The results from our transcriptomic analysis and Q‐PCR both demonstrated that there is a significant decrease in CD36 expression in macrophages in response to all of the Mtb strain infections, suggesting that down‐regulation of CD36 expression in macrophages might be a crucial host defence mechanism for restricting intracellular Mycobacterial replication.

The ATP10A gene belongs to the family of P‐type cation transport ATPases. Its functions include the translocation of phosphatidylcholine, and it is involved in plasma membrane dynamics. Its function in M.tb infection is not fully known. However, it has been identified as a novel biomarker for the discrimination of TB from latent TB infection individuals (LTBI).^23^ We observed decreased ATP10A expression in the virulent strain M.tb H37RV when compared to the avirulent strain M.tb H37Ra and BCG, implying that ATP10A could be used as a critical biomarker for identifying virulent strains of M.tb infection. LPL is a critical regulator of lipid and lipoprotein metabolism. It is expressed in a variety of cells and tissues and performs a variety of functions, including catalysing the hydrolysis of the triacylglycerol component of circulating chylomicrons and very‐low‐density lipoproteins, which release non‐esterified fatty acids and 2‐monoacylglycerol for tissue use.^24^ LPL is a therapeutic target for multiple types of metabolic diseases. Inhibition of LPL with small molecular compounds has been widely used in the clinic to treat obesity‐related syndromes, including diabetes and cardiovascular diseases. Here, we detected the down‐regulated expression of LPL in H37Rv‐infected macrophages, indicating that virulent strain M.tb infection would compete with the human body to utilize lipid and lipoprotein. Podoplanin (PDPN) is a mucin‐type transmembrane glycoprotein that can trigger platelet activation through the platelet‐receptor CLEC‐2. It is up‐regulated in many tumours and is involved in tumour metastasis and malignant progression. In a subset of tumour‐associated macrophages (TAMs), the highest expression of podoplanin (PDPN) is found.

In conclusion, with a comparison of the responses of different strains of M.tb infection in macrophages, we could identify the functionally characterized genes which were uniquely expressed during the process of virulent strain of M.tb infection. In contrast to H37RA or BCG, H37RV infection showed a richer transcriptional pattern of virulence factors which were linked with severe inflammatory immune response and altered metabolic pattern.

## CONFLICT OF INTEREST

The authors declare no conflict of interest.

## AUTHOR CONTRIBUTION

**Wenyuan Pu:** Conceptualization (equal); Data curation (equal); Formal analysis (equal); Investigation (equal); Methodology (equal); Validation (equal); Visualization (equal); Writing‐original draft (equal); Writing‐review & editing (equal). **Chen Zhao:** Data curation (equal); Formal analysis (equal). **Junaid Wazir:** Writing‐review & editing (supporting). **Mengyuan Niu:** Data curation (supporting); Formal analysis (supporting). **Zhonglan Su:** Data curation (supporting). **Shiyu Song:** Conceptualization (supporting); Data curation (supporting); Formal analysis (supporting); Methodology (supporting). **Lulu Wei:** Software (supporting). **Li Li:** Methodology (supporting). **Xia Zhang:** Supervision (supporting). **Xudong Shi:** Supervision (supporting). **Hong‐wei Wang:** Conceptualization (lead); Funding acquisition (lead); Project administration (lead); Supervision (lead); Writing‐review & editing (lead).

## Supporting information

Fig S1

Fig S2

Table S1‐S6

## Data Availability

The data that support the findings of this study are openly available in GEO reference number GSE162729.
