# Supplementary figures and images for: Comparative transcriptomic analysis of THP‐1‐derived macrophages infected with Mycobacterium tuberculosis H37Rv, H37Ra and BCG
Source: J Cell Mol Med. 2021 Oct 10;25(22):10504–20. doi: 10.1111/jcmm.16980 (PMC8581329; doi:10.1111/jcmm.16980)

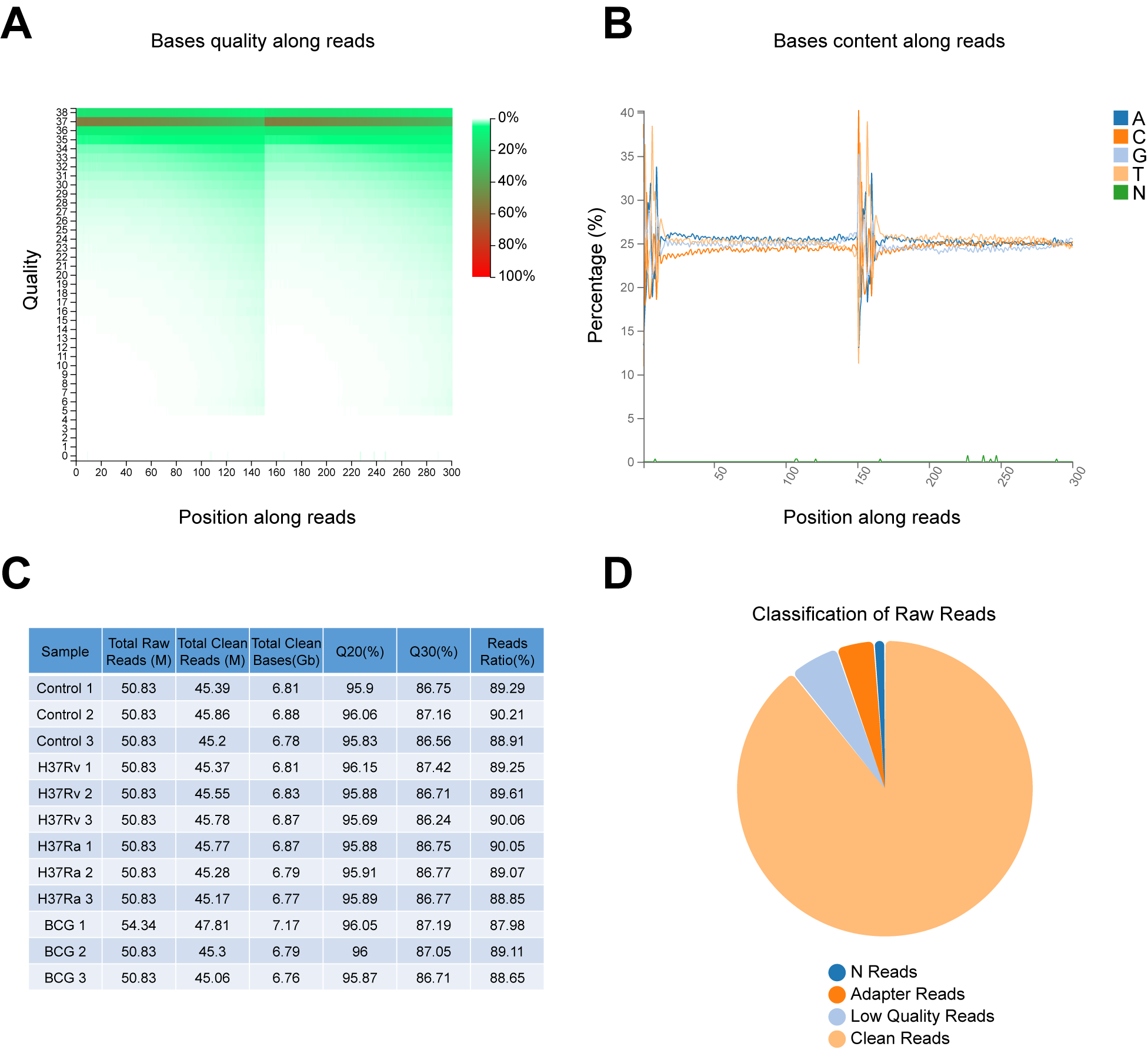

Supplement: Supplementary file 1 — Fig S1 [file JCMM-25-10504-s002.tif]

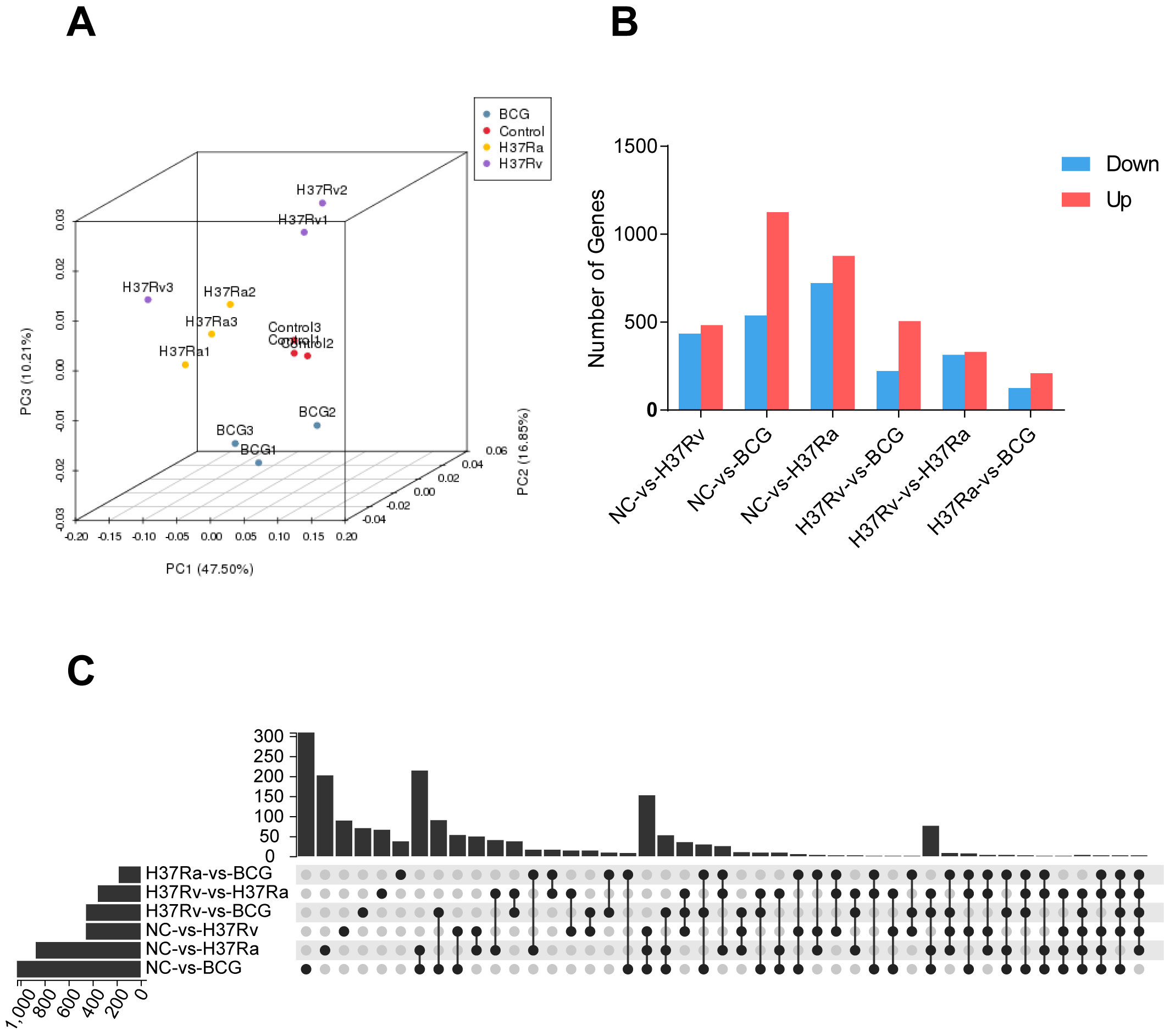

Supplement: Supplementary file 2 — Fig S2 [file JCMM-25-10504-s003.tif]
