# Supplementary material for: Comparative transcriptomic analysis of THP‐1‐derived macrophages infected with Mycobacterium tuberculosis H37Rv, H37Ra and BCG
Source: J Cell Mol Med. 2021 Oct 10;25(22):10504–20. doi: 10.1111/jcmm.16980 (PMC8581329; doi:10.1111/jcmm.16980)
Supplement: Supplementary file 3 — Table S1‐S6 [file JCMM-25-10504-s001.pdf]

Supplementary Table 1 NC vs H37Rv differentially expressed genes

Gene ID	Gene Symbol	Control Expression	Control Expression	Control Expression	H37Rv Expression	H37Rv Expression	H37Rv Expression	log_2 FC H37Rv/NC
4495	MT1G	1.14	2.07	1.91	213.46	221.4	237.65	7.05441
4493	MT1E	7.93	9.05	6.56	183.41	200.85	248.17	4.72894
4501	MT1X	17.38	20.53	22.14	370.87	386.49	600.71	4.48037
4502	MT2A	50.62	51.69	51	897.59	1033.29	1315.95	4.39469
482	ATP1B2	0.37	0.38	0.35	7.55	7.08	7.58	4.30972
5055	SERPINB2	1.12	2.01	1.8	24.28	24.49	11.37	3.68259
4494	MT1F	3.03	4.43	4.89	32.32	36.74	27.12	3.10824
4884	NPTX1	1.05	0.94	0.74	5.59	5.64	13.27	3.0404
9437	NCR1	0.14	0.25	0.31	1.57	1.79	2.25	3.02636
9945	GFPT2	0.39	0.29	0.46	2.88	3.06	1.53	2.7719
4256	MGP	9.42	9.14	9.19	0.6	0.67	0.99	-3.50779
358	AQP1	3.88	3.98	3.86	0.34	0.55	0.33	-3.24084
4332	MNDA	1.8	1.39	1.49	0.33	0.13	0.14	-2.94977
10563	CXCL13	1.74	1.7	1.14	0.25	0.15	0.21	-2.92217
8685	MARCO	3.41	3.23	3.65	0.65	0.44	0.35	-2.88523
255061	TAC4	6.04	4.71	5.92	0.87	0.75	0.75	-2.8118
3381	IBSP	1.8	1.95	1.74	0.29	0.11	0.45	-2.76324
11326	VSIG4	3.11	2.94	3.18	0.64	0.48	0.3	-2.69872
91543	RSAD2	1.38	1.53	1.07	0.23	0.19	0.11	-2.61633
7850	IL1R2	32.85	36.26	34.26	7.51	5.25	3.55	-2.58408

Supplementary Table 2 NC vs H37Ra differentially expressed genes

Gene ID	Gene Symbol	Control Expression	Control Expression	Control Expression	H37Ra Expression	H37Ra Expression	H37Ra Expression	log_2 FC H37Ra/NC
4495	MT1G	1.14	2.07	1.91	220.76	217.65	156.79	6.88497
4493	MT1E	7.93	9.05	6.56	200.94	210.31	148.91	4.58357
482	ATP1B2	0.37	0.38	0.35	8.94	6.86	10.97	4.57244
4501	MT1X	17.38	20.53	22.14	510.73	516.52	335.1	4.53317
4502	MT2A	50.62	51.69	51	1022.85	1079.38	748.54	4.23757
4884	NPTX1	1.05	0.94	0.74	9.87	10.87	5.96	3.25702
9437	NCR1	0.14	0.25	0.31	1.74	1.67	2.12	2.96096
5055	SERPINB2	1.12	2.01	1.8	7.67	10.99	15.1	2.75156
4494	MT1F	3.03	4.43	4.89	27.73	20.23	21.11	2.65306
335	APOA1	1.51	1.57	1.52	7.11	9.81	7.14	2.48951
358	AQP1	3.88	3.98	3.86	0.24	0.38	0.26	-3.69808
4256	MGP	9.42	9.14	9.19	0.57	0.61	1.05	-3.53989
255061	TAC4	6.04	4.71	5.92	0.2	0.62	0.73	-3.40367
366	AQP9	1.54	1.4	1.47	0.12	0.03	0.21	-3.23309
10563	CXCL13	1.74	1.7	1.14	0.15	0.05	0.31	-3.17041
151	ADRA2B	6.4	5.7	6.27	0.54	1.05	0.91	-2.88113
3381	IBSP	1.8	1.95	1.74	0.41	0.11	0.23	-2.87398
2006	ELN	2.43	2.47	2.7	0.22	0.53	0.27	-2.87392
8685	MARCO	3.41	3.23	3.65	0.54	0.67	0.38	-2.70319
23769	FLRT1	1.1	1.09	1.12	0.19	0.15	0.14	-2.66564

Supplementary Table 3 NC vs BCG differentially expressed genes

Gene ID	Gene Symbol	Control Expression	Control Expression	Control Expression	BCG Expression	BCG Expression	BCG Expression	log_2 FC BCG/NC
4495	MT1G	1.14	2.07	1.91	180.34	118.79	140.04	6.45759
482	ATP1B2	0.37	0.38	0.35	8.65	9.37	11.38	4.69466
4501	MT1X	17.38	20.53	22.14	467.37	350.33	345	4.31341
4493	MT1E	7.93	9.05	6.56	178.38	143.05	136.71	4.29517
4502	MT2A	50.62	51.69	51	1125.13	875.4	818.68	4.23192
4884	NPTX1	1.05	0.94	0.74	17.96	14.69	13.39	4.04416
169966	TENT5D	0.12	0	0	1.24	1.38	0.74	3.96517
9437	NCR1	0.14	0.25	0.31	2.58	1.38	3.47	3.47553
5055	SERPINB2	1.12	2.01	1.8	12	12.06	11.92	2.88697
4494	MT1F	3.03	4.43	4.89	27.9	27.29	18.63	2.72942
255061	TAC4	6.04	4.71	5.92	0.22	0.11	0.65	-4.04425
2006	ELN	2.43	2.47	2.7	0.19	0.18	0.22	-3.69062
269	AMHR2	1.21	1.71	1.47	0.21	0.14	0.1	-3.52794
284106	CISD3	1.86	2.28	1.99	0.1	0	0.56	-3.25241
358	AQP1	3.88	3.98	3.86	0.37	0.63	0.32	-3.1471
11326	VSIG4	3.11	2.94	3.18	0.23	0.41	0.23	-3.11836
8685	MARCO	3.41	3.23	3.65	0.25	0.63	0.4	-3.03695
445329	SULT1A4	2.12	1.28	2.57	0	0.76	0	-2.97193
6286	S100P	1.36	2.78	3.07	0.05	0.51	0.37	-2.96205
23397	NCAPH	20.43	20.92	21.46	2.04	3.5	2.94	-2.86189

Supplementary Table 4 Common differentially expressed genes

Gene ID	Gene Symbol	Control Expression	H37Rv Expression	H37Ra Expression	BCG Expression
219833	C11orf45	2.827	1.203	1.023	0.613
9447	AIM2	3.01	1.1	0.777	0.513
929	CD14	12.33	5.213	3.593	2.333
23397	NCAPH	20.937	5.977	5.283	2.827
1536	CYBB	2.763	1.1	1.05	0.687
6641	SNTB1	23.023	10.357	10.993	6.677
2335	FN1	406.41	135.703	144.08	71.197
6752	SSTR2	5.553	2.173	2.37	1.413
64881	PCDH20	1.98	0.863	0.58	0.553
9214	FCMR	50.26	22.287	18.177	17.07
3119	HLA-DQB1	2.673	1.163	1.013	0.877
4599	MX1	20.14	5.737	4.62	4.683
2006	ELN	2.533	0.433	0.34	0.197
255061	TAC4	5.557	0.79	0.517	0.327
7079	TIMP4	3.113	1.253	1.12	1.05
3959	LGALS3BP	38.197	17.723	16.02	15.05
4938	OAS1	31.753	11.35	9.477	8.92
11009	IL24	28.04	12.28	6.833	10.01
55509	BATF3	3.44	1.437	0.913	1.177
57214	CEMIP	4.073	1.44	0.713	1.177
26471	NUPR1	11.67	4.71	4.01	4.96
3428	IFI16	28.01	11.52	9.17	11.55
4939	OAS2	31.89	11.843	9.367	11.733
7099	TLR4	2.287	0.65	0.473	0.657
5473	PPBP	64.557	19.517	10.63	17.86
1827	RCAN1	66.447	21.787	14.647	19.457
285386	TPRG1	3.273	1.177	0.903	1.07
259307	IL4I1	58.287	22.803	18.573	21.203
115207	KCTD12	10.073	4.697	3.57	4.14
84868	HAVCR2	24.317	11.267	9.253	10.18
151	ADRA2B	6.123	1.673	0.833	1.227
1844	DUSP2	194.293	82.603	62.703	70.747
3575	IL7R	8.083	3.553	2.733	3.923
5265	SERPINA1	10.243	3.837	2.833	4.25
84171	LOXL4	2.78	0.837	0.593	1.027
1230	CCR1	51.53	20.643	16.993	23.123
1511	CTSG	3.863	1.213	1.257	1.893
83758	RBP5	6.517	1.647	1.463	2.51

122953	JDP2	4.987	1.987	1.783	2.45
374618	TEX9	9.803	2.837	2.3	3.967
4481	MSR1	7.937	1.727	1.573	1.647
3437	IFIT3	26.463	8.707	8.04	8.893
358	AQP1	3.907	0.407	0.293	0.44
51700	CYB5R2	4.077	1.253	1.253	1.3
100129969	FAM205C	6.97	2.767	2.823	2.763
94240	EPSTI1	5.807	1.247	1.327	1.327
3434	IFIT1	8.71	2.087	2.163	2.15
29968	PSAT1	108.49	29.027	29.403	29.68
115362	GBP5	4.437	2.037	2.163	2.107
10561	IFI44	17.893	4.88	5.3	5.44
3433	IFIT2	3.62	1.103	1.377	1.177
2273	FHL1	8.483	3.653	4.16	3.9
5359	PLSCR1	39.867	13.107	13.903	12.79
8685	MARCO	3.43	0.48	0.53	0.427
163732	CITED4	143.023	64.967	67.32	61.757
115265	DDIT4L	18.183	6.33	6.82	6.06
54414	SIAE	2.2	0.393	0.597	0.73
54453	RIN2	8.57	3.45	4.313	4.287
3429	IFI27	25.987	5.64	5.287	6.52
26227	PHGDH	30.01	7.047	6.853	8.27
24138	IFIT5	2.37	0.933	0.87	0.997
8519	IFITM1	18.21	3.167	2.597	3.827
256586	LYSMD2	4.36	1.29	1.4	1.597
11274	USP18	18.193	7.34	7.843	8.89
440	ASNS	31.99	8.22	8	11.433
4256	MGP	9.25	0.753	0.743	1.88
3784	KCNQ1	4.397	1.653	1.67	2.01
7850	IL1R2	34.457	5.437	5.71	8.86
8638	OASL	7.253	1.997	2.013	2.57
8743	TNFSF10	4.3	1.19	1.213	1.5
3759	KCNJ2	3.73	0.783	0.753	1.08
80162	PGGHG	7.44	0.727	3.053	2.043
6286	S100P	2.403	0.5	0.847	0.31
9235	IL32	7.44	2.5	3.513	1.783
22921	MSRB2	6.687	3.107	3.737	3.127
4973	OLR1	6.537	1.803	2.757	2.047
10346	TRIM22	7.993	3.12	3.71	2.707
11326	VSIG4	3.077	0.473	0.56	0.29
10578	GNLY	106.64	39.02	41.07	33.76
58191	CXCL16	29.643	13.247	13.677	10.967
53840	TRIM34	4.89	2.033	2.213	1.793

10993	SDS	94.507	27.283	33.067	21.193
122622	ADSSL1	1.323	3.54	3.057	4.92
5352	PLOD2	2.463	7.803	6.293	11.04
2821	GPI	45.217	105.383	99.527	138.26
390940	PINLYP	171.773	420.567	390.98	523.847
10381	TUBB3	1.28	2.897	2.627	5.067
79948	PLPPR3	6.33	12.793	16.423	23.397
91947	ARRDC4	5.923	19.71	21.087	28.813
6535	SLC6A8	53.207	128.253	142.367	202.92
402415	XKRX	1.383	3.82	3.067	4.097
8111	GPR68	5.3	15.663	12.803	17.41
440695	ETV3L	4.597	20.863	16.363	23.417
84803	GPAT3	2.163	6.74	5.563	6.8
3162	HMOX1	133.647	422.473	313.02	399.74
230	ALDOC	21.74	51.713	61.633	61.31
6319	SCD	153.997	346.883	403.997	393.913
6843	VAMP1	3.593	6.92	8.67	8.39
113177	IZUMO4	2.077	5.307	6.647	6.957
342667	STAC2	1.67	7.087	8.177	9.443
10628	TXNIP	82.317	302.963	361.73	451.323
387763	C11orf96	2.04	4.88	5.88	6.737
10105	PPIF	81.26	168.973	163.09	189.097
7053	TGM3	10.93	22.537	23.71	25.277
30001	ERO1A	17.99	41.767	45.44	45.953
51129	ANGPTL4	1.5	8.927	3.853	3.8
5055	SERPINB2	1.643	20.047	11.253	11.993
126669	SHE	1.217	2.743	2.283	2.21
10666	CD226	2.453	7.587	5.907	6.43
4494	MT1F	4.117	32.06	23.023	24.607
79870	BAALC	1.387	4.423	3.823	2.85
335	APOA1	1.533	7.517	8.02	5.013
8349	HIST2H2BE	6.607	22.04	25.393	14.14
29923	HILPDA	4.447	16.083	21.913	15.223
860	RUNX2	0.973	2.457	2.967	2.313
25976	TIPARP	18.09	48.413	54.53	50.103
7434	VIPR2	1.647	3.737	4.16	3.66
5165	PDK3	2.103	4.62	6.067	5.31
3638	INSIG1	113.403	227.29	271.617	234.033
4502	MT2A	51.103	1082.277	950.257	939.737
9351	SLC9A3R2	1.877	4.227	4	4.34
6839	SUV39H1	0.98	2.783	2.66	2.853
11015	KDELR3	6.597	12.88	13.333	13.167
2204	FCAR	9.203	20.003	19.65	19.923

6518	SLC2A5	4.04	15.113	15.723	13.5
4501	MT1X	20.017	452.69	454.117	387.567
3012	HIST1H2AE	1.04	4.27	4.283	3.47
3006	HIST1H1C	29.85	66.997	70.56	60.273
4495	MT1G	1.707	224.193	198.4	146.39
4493	MT1E	7.847	210.81	186.72	152.713
23327	NEDD4L	1.463	4.637	4.137	3.787

Supplementary Table 5 H37Rv differentially expressed genes

Gene ID	Gene Symbol	Control Expression	H37Rv Expression	H37Ra Expression	BCG Expression
57194	ATP10A	1.55	0.457	0.523	0.767
131566	DCBLD2	1.453	3.403	2.11	2.443
201799	TMEM154	1.603	0.597	0.717	0.873
650	BMP2	7.077	3.487	3.47	3.56
121512	FGD4	3.217	1.29	1.547	1.897
84824	FCRLA	5.523	15.057	10.797	4.657
1809	DPYSL3	2.1	5.553	2.107	2.313
2766	GMPR	17.587	8.477	10.753	10.063
8539	API5	1.577	4.433	6.58	3.333
64762	GAREM1	1.09	0.433	0.577	0.437
151056	PLB1	1.317	2.74	2.357	1.72
79412	KREMEN2	1.113	0.537	0.853	0.857
64866	CDCP1	3.427	6.213	3.297	4.93
166012	CHST13	1.373	0.54	0.687	0.897
2247	FGF2	1.273	2.517	1.767	1.893
64092	SAMSN1	2.233	1.177	1.513	1.493
2805	GOT1	1.537	0.683	1.327	1.667
1917	EEF1A2	27.423	13.5	14.653	17.347
4023	LPL	664.763	306.883	346.233	337.793
3209	HOXA13	1.34	0.497	0.62	0.787
51363	CHST15	6.073	12.797	11.727	6.38
7124	TNF	7.553	16.23	9.31	11.433
9180	OSMR	1.553	0.387	0.277	0.333
51191	HERC5	4.39	2.11	2.717	2.787
2920	CXCL2	13.173	39.47	20.953	27.307
100526842	RPL17-C18orf32	1.62	0.78	0.67	0.97
58489	ABHD17C	4.437	2.073	2.343	2.7
56241	SUSD2	1.49	0.37	0.627	0.983
3207	HOXA11	4.837	2.427	2.833	2.967
387522	TMEM189-UBE2V1	2.047	5.597	2.343	3.453
2921	CXCL3	24.39	51.957	27.713	38.73
83690	CRISPLD1	1.387	0.613	0.957	1.077
8614	STC2	1.393	0.74	0.643	1.027
8651	SOCS1	1.513	0.657	0.953	0.917
338657	CCDC84	3.767	6.227	5.743	6.13
100132247	NPIPB5	1.08	2.17	1.48	1.633

105369230	105369230	4.397	1.913	3.013	2.043
6241	RRM2	1.483	3.153	2.08	2.31
1545	CYP1B1	24.237	61.457	46.307	43.193
25841	ABTB2	2.263	4.583	4.127	4.18
6236	RRAD	15.503	29.477	13.803	16.48
27161	AGO2	2.02	4.26	2.713	3.78
2919	CXCL1	20.727	51.807	19.777	27.643
1018	CDK3	1.087	0.223	0.617	0.807
3274	HRH2	1.737	0.627	0.707	1.133
10669	CGREF1	1.717	0.833	1.227	1.03
3777	KCNK3	2.537	5.727	5.033	4.143
1475	CSTA	2.293	0.85	1.053	1.207
89848	FCHSD1	14.61	31.727	30.71	26.303
6855	SYP	11.38	21.89	18.807	15.11
495	ATP4A	1.457	11.087	1.493	1.457
22901	ARSG	2.98	6.343	6.133	5.387
8642	DCHS1	1.743	3.69	2.79	2.67
3134	HLA-F	1.043	0.327	0.507	0.443
745	MYRF	15.85	30.84	29.913	28.563
10630	PDPN	2.797	1.293	1.517	1.62
57605	PITPNM2	0.933	1.83	1.563	1.65
102724560	CBSL	3.8	1.717	1.917	2.44

Supplementary Table 6 Primers for real-time polymerase chain reaction (PCR) analysis

Genes	Forward Primer (5′–3′)	Reverse Primer (5′–3′)
IL1β	AAATACCTGTGGCCTTGGGC	TTTGGGATCTACACTCTCCAGCT
TNF-α	GAGGCCAAGCCCTGGTATG	CGGGCCGATTGATCTCAGC
IL10	GACTTTAAGGGTTACCTGGGTTG	TCACATGCGCCTTGATGTCTG
CCL2	CTTCTGTGCCTGCTGCTCAT	CGGAGTTTGGGTTTGCTTGTC
CCL3	GCTCTCTGCAACCAGTTCTCTG	TTCTGGACCCACTCCTCACTG
CCL4	CTGTGCTGATCCCAGTGAATC	TCAGTTCAGTTCCAGGTCATACA
CD14	ACGCCAGAACCTTGTGAGC	GCATGGATCTCCACCTCTACTG
CD36	GGCTGTGACCGGAACTGTG	AGGTCTCCAACTGGCATTAGAA
TLR4	AGACCTGTCCCTGAACCCTAT	CGATGGACTTCTAAACCAGCCA
CXCL8	AGACAGCAGAGCACACAAGC	ATGGTTCCTTCCGGTGGT
CSF2	CATGATGGCCAGCCACTACAA	ACTGGCTCCCAGCAGTCAAAG
IRF9	GCCCTACAAGGTGTATCAGTTG	TGCTGTCGCTTTGATGGTACT
